# Nitrate Derived From Beetroot Juice Lowers Blood Pressure in Patients With Arterial Hypertension: A Systematic Review and Meta-Analysis

**DOI:** 10.3389/fnut.2022.823039

**Published:** 2022-03-15

**Authors:** Cicero Jonas R. Benjamim, Andrey Alves Porto, Vitor Engrácia Valenti, Andressa Crystine da Silva Sobrinho, David M. Garner, Bruno Gualano, Carlos Roberto Bueno Júnior

**Affiliations:** ^1^Department of Internal Medicine, Ribeirao Preto Medical School, University of São Paulo, Ribeirão Preto, Brazil; ^2^Autonomic Nervous System Center, UNESP, Marilia, Brazil; ^3^Cardiorespiratory Research Group, Department of Biological and Medical Sciences, Faculty of Health and Life Sciences, Oxford Brookes University, Oxford, United Kingdom; ^4^Applied Physiology and Nutrition Research Group, School Medicine, University of São Paulo, São Paulo, Brazil; ^5^School of Physical Education of Ribeirão Preto, University of São Paulo, Ribeirão Preto, Brazil

**Keywords:** dietary supplementation, *Beta vulgaris* L., nitrates, nitric oxide, hypertension

## Abstract

**Background:**

Although there are a considerable number of clinical studies on nitrate (NO_3_) rich beetroot juice (BRJ) and hypertension, it is difficult to indicate the real effects of NO_3_ from BRJ on the BP of hypertensive patients because there are still no estimates of the effects of NO_3_ derived from BRJ on the BP of hypertension patients.

**Objective:**

To clarify these effects, we developed a systematic literature review with a meta-analysis of randomized clinical trials (RCTs).

**Design:**

The searches were accomplished through EMBASE, Cochrane Library, MEDLINE, CINAHL, Web of Science, and LILACS databases. The study included single or double-blinded RCTs and participants older than 18 years with hypertension [systolic BP (SBP) > 130 mmHg and diastolic BP (DBP) > 80 mmHg]. NO_3_ BRJ was required to be consumed in a format that possibly blinded participants/researchers. These studies should also report the SBP and DBP values (mmHg) measured before and after the treatment. Risk of Bias tools and GRADE were enforced.

**Results:**

Seven studies were included (218 participants). BRJ intervention time ranged from 3 to 60 days with daily dosages of 70–250 mL of BRJ. After the intervention with NO_3_ from BRJ, SBP underwent significant changes (*p* < 0.001) of −4.95 (95% CI: −8.88; −1.01) (GRADE: ⊕⊕⊕○ Moderate), but not for DBP (*p* = 0.06) −0.90 mmHg (95% CI: −3.16; 1.36) (GRADE: ⊕⊕⊕○ Moderate), compared to the control group.

**Conclusions:**

The NO_3_ derived from BRJ reduces SBP, but not DBP in patients with arterial hypertension.

**Systematic Review Registration:**

https://www.crd.york.ac.uk/prospero/display_record.php?RecordID=269339.

## Introduction

Beetroot juice (BRJ) is rich in nitrate (NO_3_) and has the potential to reduce blood pressure (BP). NO_3_ is a precursor for the production of nitric oxide (NO) and increases its concentrations in the bloodstream, optimizing endothelial function (e.g., vasodilation) (1). A recent meta-analysis found that BRJ NO_3_ (2–56 days of intervention) reduced typically −3.55 mmHg and −1.32 mmHg for systolic (SBP) and diastolic BP (DBP), respectively, in a mixed sample of individuals with and without arterial hypertension (2). This result is of clinical relevance in the control of arterial hypertension, as a 2-mmHg reduction in BP can reduce mortality from ischemic heart disease by 7 and 10% of mortality from stroke (3). The latest systematic review with only hypertensive patients concluded that there is insufficient evidence to support or refute the use of inorganic NO_3_ as a strategy to decrease BP. However, this review included studies intervening with NO_3_ salts not from beetroot only and physical exercise programs, lacking an estimate of the effect of the intervention. Therefore, there are significant limitations to the conclusions on the influence of BRJ in hypertensive patients restricted to its findings (4).

Beetroot is rich in bioactive compounds (betalains, flavonoids, and polyphenols), which can influence the endothelial and pressure responses differently from NO_3_ salts (5). Although there are a considerable number of clinical studies on BRJ rich in NO_3_ and hypertension, it is difficult to indicate the real effects of NO_3_ from BRJ on the BP of hypertensive patients because there are still no estimates of the effects of NO_3_ derived from BRJ on the BP of patients with hypertension. Based on the information presented above, we raise the following question: is the NO_3_ of the BRJ capable of decreasing the BP in hypertensive patients? To clarify this issue, our study aimed to carry out a systematic review with meta-analysis to verify the effects of the NO_3_ of the BRJ on the BP of patients with hypertension and to include subanalyses with BP values obtained by clinical measurements and ambulatory 24-h monitoring.

## Materials and Methods

### Registration

The review was described according to the recommendations of the Preferred Reporting Items for Systematic Reviews and Meta-Analyzes (PRISMA) (6) and is registered in the PROSPERO database (CRD42021269339).

### Search Strategy and Study Selection

The searches were accomplished through EMBASE, Cochrane Library, MEDLINE (via PubMed), CINAHL, Web of Science, and LILACS databases with the application of the keywords “Beetroot juice” OR “Nitrates” OR “Red beet” OR “Beta vulgaris” AND “Blood pressure” OR “Hypertension.” The search relied on the Boolean NOT for the descriptor “Exercise.”

All articles identified were exported to the Rayyan QCRI program (Qatar Computing Research Institute, Qatar) to exclude duplicates. The studies were screened in the Rayyan program by reading the title and abstract. The eligibility stage was achieved by reading the articles entirely by two independent reviewers (CJRB and AAP). Another reviewer was invited to give a judgment (VEV) if there was a difference of opinion concerning a study.

The studies were required to originate from peer-reviewed journals published from the interception of the database until July 14, 2021. For inclusion, the articles needed to accomplish all the criteria are described below as follows: single or double-blinded RCTs design; participants older than >18 years old, previously diagnosed with hypertension or higher BP inclusion criteria for baseline SBP > 130 mmHg and DBP > 80 mmHg in accordance to American College of Cardiology (ACC) and American Heart Association (AHA) (7). NO_3_ from BRJ should necessarily be consumed in a format that blinded participants and researchers, and control intervention should use BRJ in a NO_3_ depleted condition. These studies reported the SBP and DBP values (mmHg) were measured before and after the intervention.

### Data Extraction

Information about the author, study design, features of the study participants, intervention, and the results of the respective studies were reported. Missing data were requested by contacting the corresponding study authors. This stage was completed independently by one reviewer (CJRB). When the author's correspondent did not answer, the Web Plot Digitizer® was applied to extract data presented in the graphs. We adopted the criteria of extracting the data postintervention, and the mean and standard deviations (MSD) were logged. Values presented with “standard error” or “confidence intervals” (CI) in the studies were transformed to MSD.

### Assessment of the Risk of Bias

The bias analysis was completed at Risk of Bias tools originated in the Cochrane organization (8) *via* the Review Manager program (RevMan 5.4.1). Risk of bias is a tool founded on the domains (9). The evaluation was split into seven fields: “Random sequence generation,” “Allocation concealment,” “Blinding of participants and personnel,” “Blinding of outcome assessment,” “Incomplete outcome data,” “Selective reporting,” and “Other Bias.” The classification was split into three direct responses: low risk, unclear risk, and high risk. Our deductions were based on the table developed by Carvalho et al. (9), “Reviewer's judgment and criteria for judgment.” Two independent authors achieved the analysis of the risk of bias (CJRB & AAP) and a third (VEV) was consulted if there were any discrepancies in the decisions.

### GRADE (Levels of Evidence)

The Grades of Recommendation, Assessment, Development, and Evaluation (GRADE) Working Group (GRADE Working Group, 2004) was surveyed to analyze the certainty of the evidence, including the study design of randomized trials (strong evidence). Study quality (detailed study methods and execution) and significant limitations secondarily were considered in the strength of evidence analysis (10). The summary of the findings table was created using GRADEpro GDT version 4® (McMaster University, ON, Canada).

### Qualitative Analysis (Systematic Review)

A narrative synthesis was executed to describe detailed data on how each study was completed. The details for each study were introduced in texts and tables. The results of the individual qualitative analysis for each study were made by analyzing the behavior of SBP and DBP (mmHg) pre- and postintervention BRJ rich in NO_3_ or without NO_3_.

### Quantitative Analysis (Meta-Analysis)

In the meta-analysis, we inserted the SBP and DBP clinical values (measured with a sphygmomanometer) and ambulatory 24 h (measuring every 15–30 min during 24-h monitoring). The effects of BRJ interventions on SBP and DBP were assessed on the alteration between the intervention and control groups. The data enforced to construct the meta-analysis was the period postintervention.

Heterogeneity was calculated *via* the *I*^2^ statistic, where a number >50% was considered to indicate substantial heterogeneity between the tests (11). For the values of “95% CI” and “Test for overall effect size,” values of *p* < 0.05 were assumed as significant differences. We enforced a random-effects model, considering that this is a more conservative method which allows that the heterogeneity of the study may fluctuate beyond chance, providing further generalizable results (8). All data was made by the Review Manager Program (RevMan 5.4.1).

## Results

**Figure 1:**
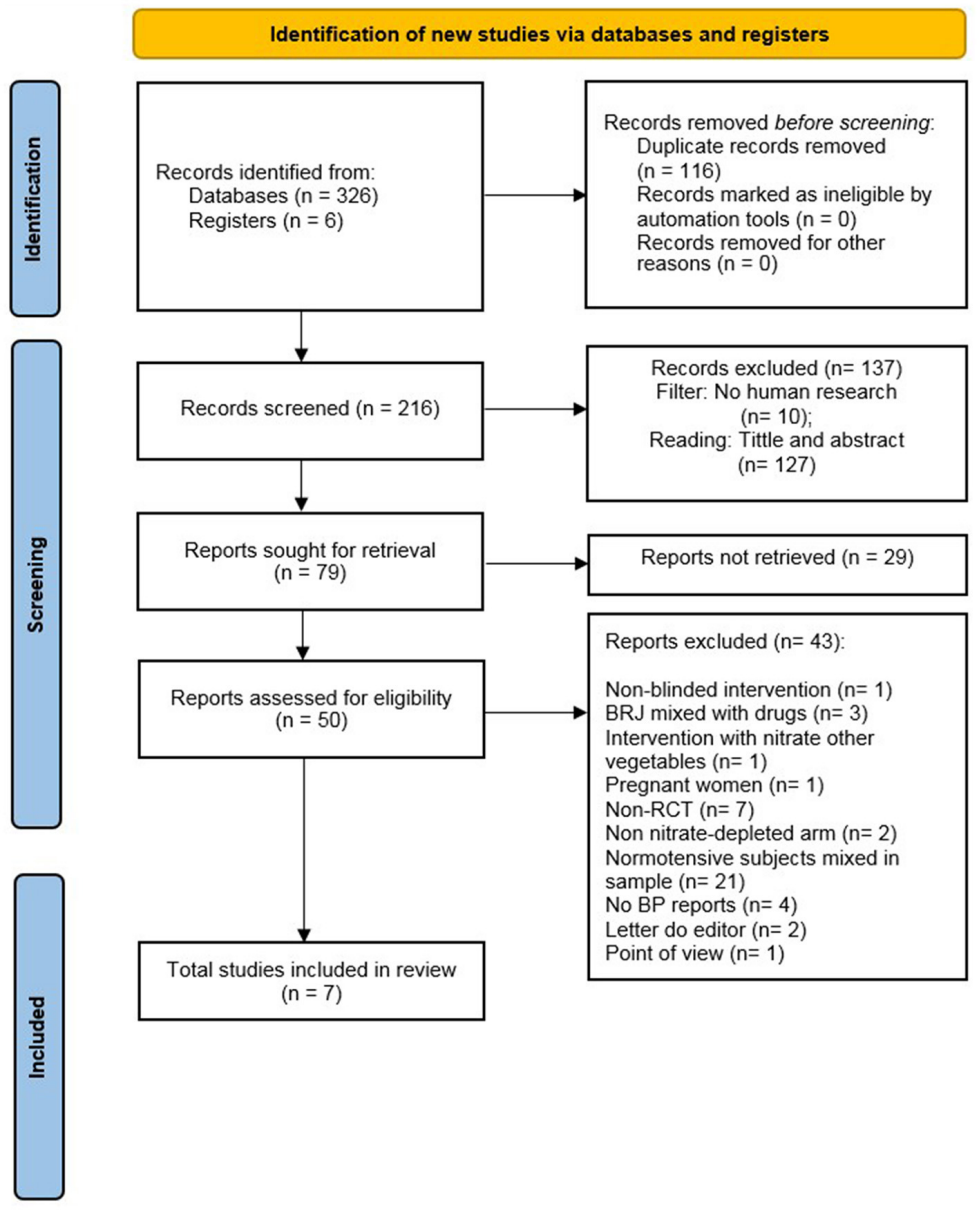
Flowchart prisma.

A total of 326 studies were identified via searches in the databases. After the removal of duplicates (*n* = 116), 210 publications were screened for inclusion. Amongst them, 137 records were excluded after reviewing the title/abstract. The remaining 79 papers were selected for full-text reading. Finally, seven studies were included in the qualitative (systematic review) and quantitative (meta-analysis) synthesis. The search process and selection step details are confirmed in the flow diagram of the PRISMA protocol (Figure 1).

The studies included in this review were published between 2013 and 2020 (Table 1). Three studies were completed in the United Kingdom (15, 16, 18), and the others were undertaken in Australia (13), Brazil (12), the USA (14), and Ireland (17).

**Table 1:** Description of the characteristics of the study population, intervention, and outcomes articles by author and year, trial design, sample and sex, age (years), BMI (kg/m^2^), BP baseline, BRJ dose/duration, BRJ NO_3_ concentration, washout period, placebo, and outcomes.

**References**	**RCT design**	**Sample and sex**	**Age (years)**	**BMI (kg/m^2^)**	**Baseline BP**		**BRJ dose/duration**	**BRJ NO3 (mmol)**	**Washout period**	**Placebo**	**Outcomes**
					**SBP (mmHg)**	**DBP**					
Baião et al. (12)	Crossover	5 F	54.25 ± 4.64	35.08 ± 2.54	164 ± 24.25	91.75 ± 6.23	60 g cereal bar produced with BRJ/3 weeks	9.5 ± 0.05	2 weeks	Cereal bar produced with BRJ NO3 depleted	NO_3_ from BRJ lowers clinic SBP reduced and DBP reduced.
Bondonno et al. (13)	Crossover	10 M and 17 F	63.2 ± 4.4	26.9 ± 3.2	132.9 ± 11.8	76.2 ± 10.4	140 mL/1 week	6.2	2 weeks	BRJ NO3 depleted	NO3 from BRJ not reduced ambulatory 24hrs SBP and DBP.
Broxterman et al. (14) (Study 1)	Counter balanced	10 M and 3 F with medication	53 ± 12	26 ± 4	130 ± 8	76 ± 12	70 mL/3 days	6.2	2 weeks	BRJ NO3 depleted	NO3 from BRJ reduces clinic SBP and DBP in patients without medication, but not in patients with medication.
Broxterman et al. (14) (Study 2)	Counter balanced	11 M and 3 F without medication	49 ± 13	27 ± 5	126 ± 15	73 ± 13					
Gilchrist et al. (15)	Crossover	18 M and 9 F	67.2 ± 4.9	30.8 ± 3.2	142.9 ± 13.9	81.1 ± 9.2	250 mL/ 2 weeks	7.5	4 weeks	BRJ NO3 depleted	NO3 from BRJ was unable to reduce ambulatory 24hrs SBP than DBP in hypertensive diabetic patients.
Kapil et al. (16)	Parallel	P: 10 M and 22 F NO3: 16 M and 16 F	P: 56.3 ± 16.4 NO3: 57.6 ± 13.9	P: 26.5 ± 4.0 NO3: 28.6 ± 5.0	P: 148.2 ± 10 NO3: 149 ± 11	P: 88.2 ± 8.0 NO3: 88.9 ± 9.8	250 mL/4 weeks	6.4	2 weeks	BRJ NO3 depleted	NO3 from BRJ reduced both clinic and ambulatory 24hrs SBP and DBP.
Kerley et al. (17)	Crossover	13 M and 7 F	62.5 ± 13.1	30.7 ± 5.8	137 ± 7	80 ± 7	140 mL/ 1 week	12.8	Not reported	BRJ NO3 depleted	NO3 from BRJ reduced ambulatory 24hrs SBP and DBP.
Siervo et al. (18)	Parallel	P: 1 M and 15 F NO3: 10 M and 5 F	P: 147 ± 14.5 NO3: 155.8 ± 18.6	P: 27.3 ± 5.7 NO3: 29.1 ± 5.8	P: 147 ± 14.5 NO3: 155.8 ± 18.6	P: 89.2 ± 9.0 NO3: 94.0 ± 10.5	70 mL/30 and 60 days	6.4	Not reported	BRJ NO3 depleted	NO3 from BRJ reduced clinic SBP in 30 and 60 days, but not DBP. Ambulatory 24hrs SBP was reduced in 60 days.

*F, female; M, masculine; BMI, body mass index; P, placebo; NO_3_, nitrate; BRJ, beetroot juice; BP, blood pressure; SBP, systolic blood pressure; DBP, diastolic blood pressure; mmHg, millimeter of mercury; mmol, milimoles; mL, milliliters; g, grams*.

All studies included in the systematic review participated in the calculation of the meta-analysis but were allocated into “Clinic” and “Ambulatory” subgroups. Only the studies by Kapil et al. (16) and Siervo et al. (18) performed both clinical and ambulatory 24-h measurements of SBP and DBP and had data entered into both the subgroups. Complementarily, we performed the calculation with the values of all clinical and ambulatory measurements together. The net deviations and 95% CI constant with the BP values for each assay are illustrated in Table 2, **Figure 3**.

**Table 2:** Summary of findings: GRADE.

**Summary of findings:**					
**BRJ rich in nitrate compared to BRJ nitrate-depleted for arterial hypertension**					
**Patient or population**: Patients with hypertension. **Intervention**: BRJ rich in nitrate **Comparison**: BRJ nitrate-depleted					
**Outcome No of participants (studies)**	**Anticipated absolute effects (95% CI)**			**Certainty**	**What happens**
	**Comparison**		**Intervention (Difference)**		
Systolic BP (Clinic) No of participants: 96 (5 RCTs)	The mean systolic BP (Clinic) was **145.69** mmHg	-	MD **7.69 mmHg lower** (15.26 lower to 0.11 lower)	⊕⊕⊕○ MODERATE Due to serious risk of bias. Due to serious inconsistency. Due to serious imprecision. Upgraded due to very large magnitude of effect. Upgraded because all plausible confounding would suggest spurious effect.	Nitrate results in a reduction in clinic blood pressure.
Diastolic BP (Clinic) No of participants: 96 (5 RCTs)	The mean diastolic BP (Clinic) was **85.6** mmHg	-	MD **1.42 mmHg lower** (5.85 lower to 3 higher)	⊕⊕⊕○ MODERATE Due to serious risk of bias. Due to serious inconsistency. Due to serious imprecision. Upgraded due to large magnitude of effect. Upgraded because all plausible confounding would reduce demonstrated effect.	Nitrate results in a slight reduction in clinic diastolic blood pressure.
Systolic BP (Ambulatory) No of participants: 122 (5 RCTs)	The mean systolic BP ambulatory 24 h was **135.91** mmHg	-	MD **2.68 mmHg lower** (5.73 lower to 0.37 higher)	⊕⊕⊕○ MODERATE Due to serious risk of bias. Due to serious inconsistency. Due to serious imprecision. Upgraded due to large magnitude of effect. Upgraded because all plausible confounding would reduce demonstrated effect.	Nitrate likely does not reduce ambulatory systolic blood pressure.
Diastolic BP (Ambulatory) No of participants: 122 (5 RCTs)	The mean diastolic BP ambulatory 24 h was **80.55** mmHg	-	MD **0.63 mmHg lower** (2.94 lower to 1.68 higher)	⊕⊕⊕○ MODERATE Due to serious risk of bias. Due to serious inconsistency. Due to serious imprecision. Upgraded due to large magnitude of effect. Upgraded because all plausible confounding would reduce demonstrated effect.	Nitrate likely does not reduce ambulatory diastolic blood pressure.
Systolic BP (both) No of participants: 218 (7 RCTs)	The mean systolic BP (both) was **141.25** mmHg	-	MD **4.95 mmHg lower** (8.88 lower to 1.01 lower)	⊕⊕⊕○ MODERATE Due to serious risk of bias. Due to serious inconsistency. Due to serious imprecision. Upgraded due to large magnitude of effect. Upgraded because all plausible confounding would reduce demonstrated effect.	Nitrate results in a large reduction in systolic blood pressure in both clinic and ambulatory values.
Diastolic BP (both) No of participants: 218 (7 RCTs)	The mean diastolic BP (both) was **83.34** mmHg	-	MD **0.9 mmHg lower** (3.16 lower to 1.36 higher)	⊕⊕⊕○ MODERATE Due to serious risk of bias. Due to serious inconsistency. Due to serious imprecision. Upgraded due to large magnitude of effect. Upgraded because all plausible confounding would reduce demonstrated effect.	Nitrate results in little to no difference in diastolic blood pressure in both clinic and ambulatory values.

*The risk in the intervention group (and its 95% CI) is based on the assumed risk in the comparison group and the relative effect of the intervention (and its 95% CI). CI, Confidence interval; MD, Mean difference*.

The study by Siervo et al. (18) was included twice in the clinical BP measurement meta-analysis graph, as the measurement of SBP and DBP occurred twice, at 30 (n-1) and 60 (n-2) days after the start of the intervention. The study by Broxterman et al. (14) took results from the two clinical trials: Study 1 was performed with patients using pharmacotherapies for arterial hypertension and Study 2 with patients without pharmacotherapies for arterial hypertension.

### Analysis of the Risk of Bias

**Figure 2:**
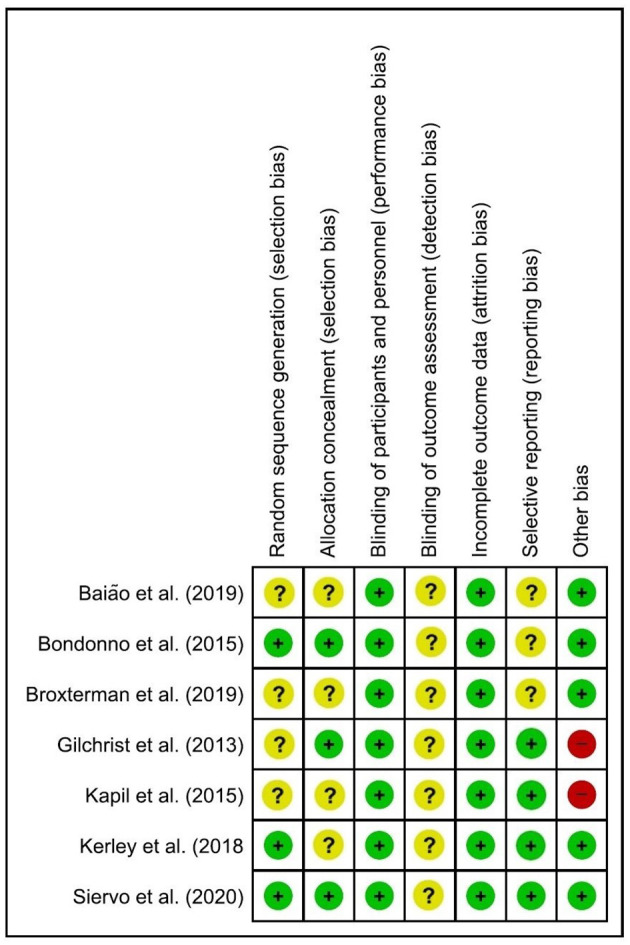
Cochrane risk of bias tool.

Most studies presented “Low risk” or “Uncertain risk” of bias according to the reviewers' judgment (Figure 2). In the domain of “Random sequence generation,” Kapil et al. (16), Baião et al. (12), Broxterman et al. (14), and Gilchrist et al. (15) did not provide information on how the random sequence of treatment was generated and presented an uncertain risk of bias. The studies by Bondonno et al. (13), Kerley et al. (17), and Siervo et al. (18) showed a low risk of bias as they generated the allocation sequence from online software. In the domain of “Allocation concealment,” Kapil et al. (16), Baião et al. (12), Broxterman et al. (14), and Kerley et al. (17) did not provide information on whether the intervention vials were coded with placebo or experimental treatment and who was responsible for this and therefore presented an uncertain risk of bias. Bondonno et al. (13), Gilchrist et al. (15), and Siervo et al. (18) presented a low risk of bias in this domain as they coded interventions, and the codes were placed in sealed letters.

In “Blinding of participants and personnel” all studies presented a low risk of bias as they concealed the experimental intervention with the BRJ without NO_3_. No studies provided information on “Blinding of outcome assessment,” and so all the studies presented an uncertain risk of bias in this domain.

In “Incomplete outcome data,” all studies presented a low risk of bias, as they presented data on all variables that were evaluated in the methodology section of the article. Regarding the “Selective reporting” domain, three of the seven studies presented an uncertain risk of bias, as they did not publish the clinical trial protocol and, it is impossible to know whether the results of all published variables were previously described in the protocols.

For the domain of “Other bias,” the study by Kapil et al. (16) and Gilchrist et al. (15) presented a high risk of bias, as they did not recommend not to use mouthwash or to use antibiotics during the experiments. The other studies (12–14, 17, 18) demonstrated a low risk of bias, as they controlled the pharmacotherapies during the study, provided a list of foods rich in nitrate for participants to avoid during the study, and provided guidelines not to use mouthwash during the study.

### BRJ Rich in Nitrate on SBP and DBP (Qualitative Results)

The intervention time with BRJ in the studies ranged from 3 to 60 days with daily dosages of 70–250 mL of BRJ. Only two studies reported the time of the day when BRJ was consumed, where Kerley et al. (17) offered BRJ at 09:00 a.m and Gilchrist et al. (15) between 06:00 and 08:00 p.m. Four included studies had crossinterventions (12, 13, 15, 17), two presented a parallel study design (16, 18) and a counterbalanced (14). Five of the seven studies established a washout period between interventions that ranged from 2 to 4 weeks (12–16) (Table 1). Studies performed blood or salivary analyzes to attest to changes in plasma nitrite/nitrate concentrations, and all studies found significant increases in nitrite/nitrate concentrations during and after intervention with NO_3_ rich BRJ (12–18).

Among five studies that accomplished BP measurements by clinical measurement, four demonstrated a reduction in SBP values (12, 14, 16, 18) and three studies established a reduction in BP DBP (12, 14, 16). Of the five studies that obtained BP values by a 24-h ambulatory measurement, three showed a significant reduction in SBP (16–18) and none of the studies showed a significant reduction in DBP. The benefits of nitrate-rich BRJ in reducing BP were less significant when 24-h ambulatory BP measurement was performed compared to clinical measurement studies. The certainty of evidence from the studies is presented in the GRADE summary of findings (Table 2).

### BRJ Rich in Nitrate on SBP and DBP Values (Quantitative Results)

We applied a random effect and mean difference model to quantify the effect size (black diamonds); the diamond dimension represents the 95% CI. A negative effect indicates decreased SBP and DBP values in the nitrate-rich BRJ group compared to placebo.

**Figure 3:**
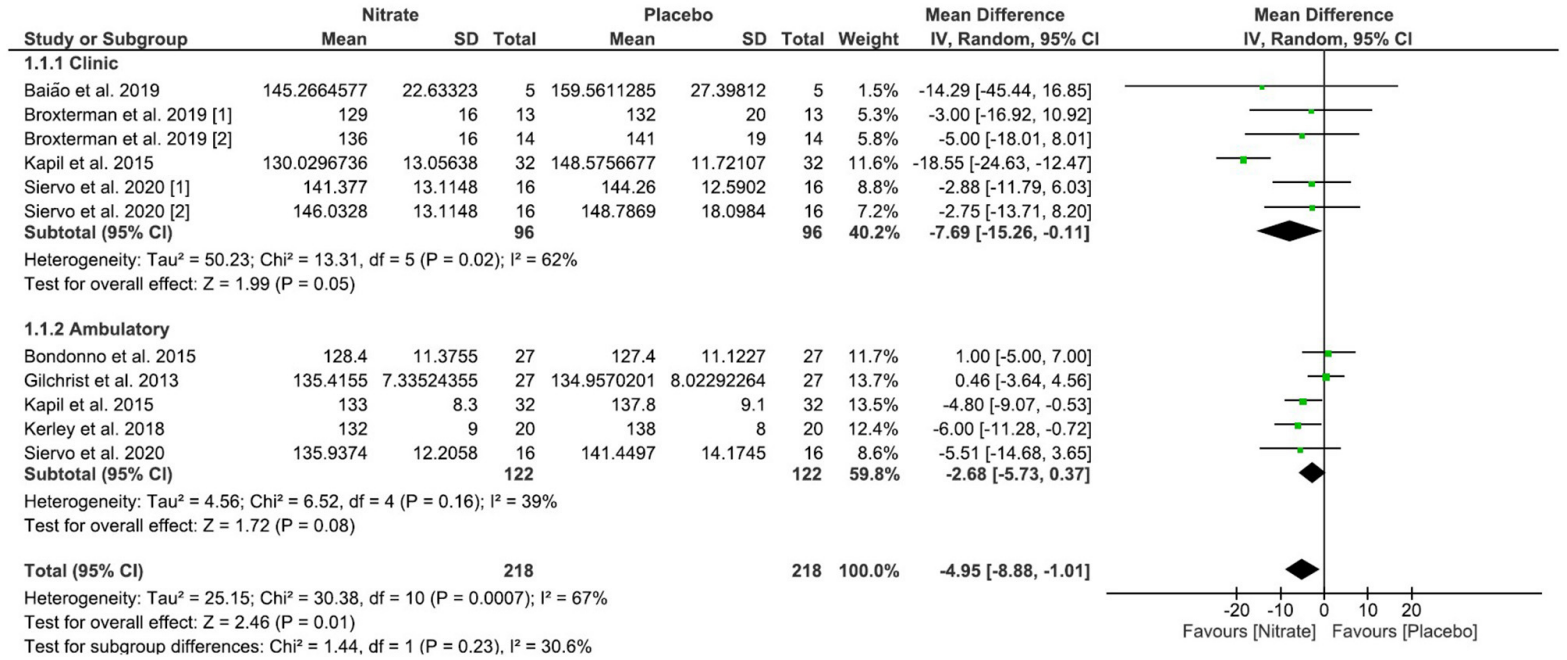
Effects of NO_3_ derived from BRJ on SBP (mmHg) values.

In clinically measured SBP values, an important decrease was observed in the “Test for overall effect,” where we revealed a *p* = 0.04 and heterogeneity of 62%. The subtotal (CI) was −7.69 mmHg (95% CI: −15.26; −0.11). In the ambulatory SBP values, no significant changes were observed in “Test for overall effect,” and we found a value of *p* = 0.16 and heterogeneity of 39%. The subtotal (CI) was −2.68 mmHg (95% CI: −5.73; 0.37). When we calculated SBP values for the two forms of BP measurement (clinical and ambulatory), significant changes were observed, in which for the “Test for overall effect” we found a *p* < 0.001 and heterogeneity of 67%. The total (CI) was −4.95 (95%CI: −8.88; −1.01) (Figure 3).

**Figure 4:**
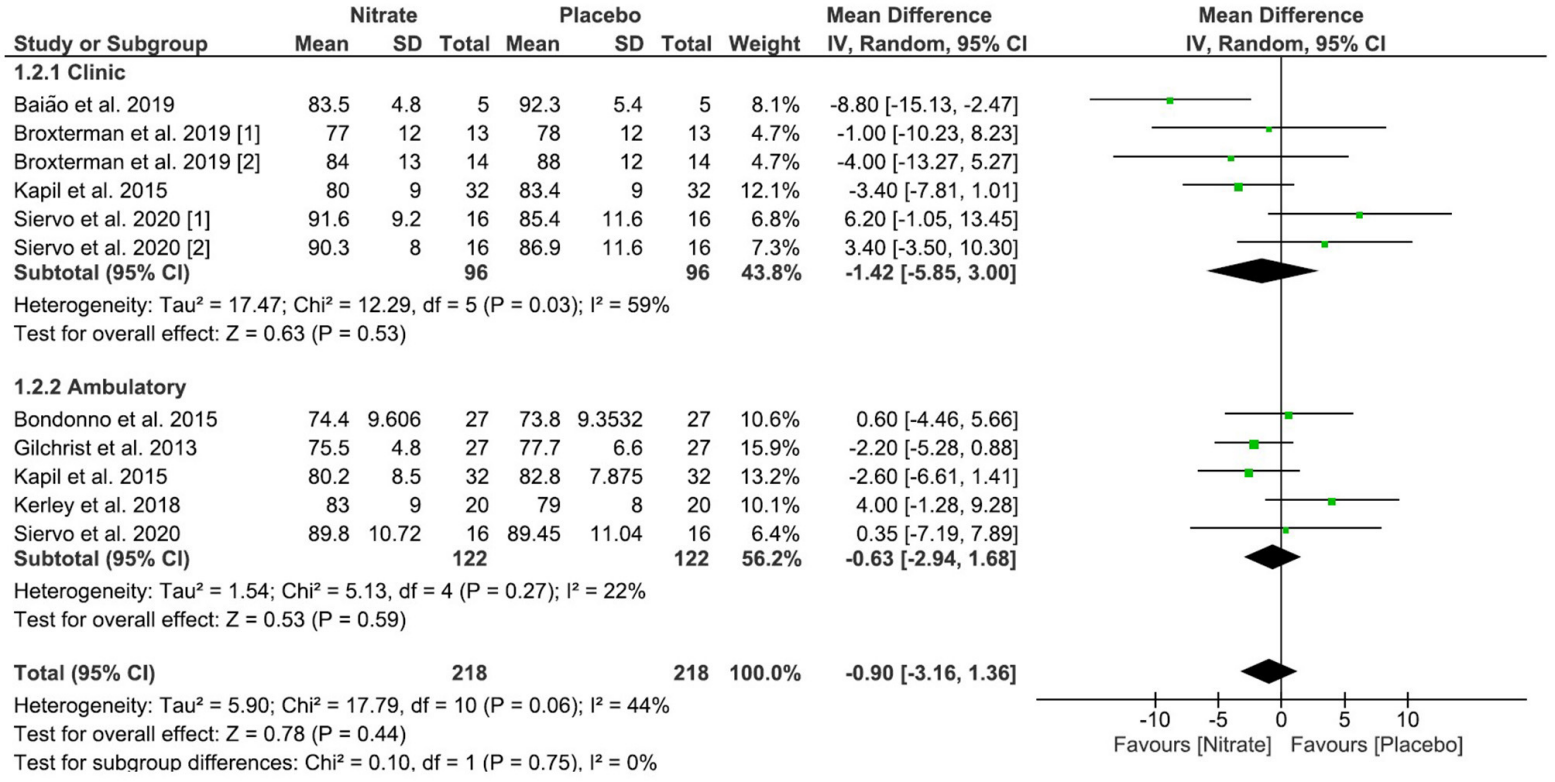
Effects of NO_3_ derived from BRJ on DBP (mmHg) values.

A significant reduction was observed for the clinical DBP values for “Test for overall effect,” which revealed a value of *p* = 0.03 and heterogeneity of 59%. The subtotal (CI) was −1.42 mmHg (95% CI: −5.85; 3.00). No significant change was observed for outpatient DBP values. For the “Test for overall effect,” we revealed a value of *p* = 0.59 and heterogeneity of 22%. The subtotal (CI) was −0.63 mmHg (95%CI: −2.94; 1.68). When outpatient and clinical DBP values were analyzed together in calculating the meta-analysis, no significant change was observed. The “Test for overall effect” revealed a value of *p* = 0.06 and heterogeneity of 44%. The subtotal CI was −0.90 mmHg (95% CI: −3.16; 1.36) (Figure 4).

Sensitivity analyzes were completed to assess the influence of the results of each study on the overall outcome of the meta-analysis (Supplementary Material 1).

## Discussion

This systematic review with meta-analysis was completed with the aim of clarifying the effects of NO_3_ of the BRJ on the SBP and DBP of patients with arterial hypertension. Our study is innovative due to the stratification of the effects of NO_3_ between BP values obtained by clinical and 24-h ambulatory measurements, and it is the first meta-analysis to analyze the effects of NO_3_ of BRJ in an exclusive sample of patients with hypertension.

The increase in NO concentration promotes vascular smooth muscle relaxation by different cellular mechanisms [e.g., activation of K+ channels; cyclic guanosine monophosphate (cGMP)-dependent protein kinase (PKG)] and then promotes blood pressure decrease through muscle relaxation in the endothelium *via* increased activity of endothelial NO synthase (eNOS) (19). NO also appears to modify neural control of the cardiovascular system by reducing the flow of sympathetic modulation (20). Through nitrate/nitrite salivary and blood tests, the studies included in this review confirmed that the decrease in SBP was due to the increase in nitrate and nitrite mediating the augment in NO production (12–18).

Beetroot juice also has antioxidant substances such as flavonoids, anthocyanins, and betaine mixed to NO_3_, which can optimize endothelial function and lower blood pressure in different pathways than isolated NO_3_ salts use (5, 21). Studies with only NO_3_ salts instead of NO_3_-rich BRJ demonstrate smaller changes in SBP (2, 22). The synergistic effects of NO_3_ from BRJ with other beverage components (e.g., betaines, anthocyanins, vitamins, and minerals) on BP are unknown, but we assume they exist. We recognized the difficulty in solving these questions, as studies should control the effects of NO_3_ from BRJ with an intervention without NO_3_ and without the bioactive compounds present in BRJ. To date, interventions that offer these characteristics are unknown.

In the subgroup analysis, we divided BP measurements into “clinical” and “ambulatory,” and the calculation of the meta-analysis revealed that the NO_3_ of the BRJ decreases the SBP of studies in which the values are obtained with clinical measurement. In studies where only ambulatory 24-h data are analyzed, the SBP is not lowered compared to the control group. The values obtained by both clinical and ambulatory measurements were added to the total calculation of the meta-analysis, and the decrease in SBP under the effects of BRJ NO_3_ remains. For DBP values, a significant reduction was only detected when this parameter was measured clinically but was not diminished when there was a 24-h ambulatory measurement. Furthermore, the analysis of clinical and outpatient values together did not show a significant reduction in DBP, reinforcing that DBP is not significantly influenced by the NO_3_ of the BRJ.

There is no explanation why the DBP was not reduced. Based on other observations, we understand that this is due to DBP having low or slight variation in studies focused on the treatment of hypertension, especially when the prevalence of hypertensive patients with baseline DBP > 90 mmHg is low, as in the RCTs included in this review (23).

Regarding SBP, the study by Banegas et al. (24), with a sample of 1,04,639 hypertensive patients, demonstrated that its values when obtained by clinical measurement are ~12 mmHg higher than values with 24-h ambulatory measurement, and this study elucidates the importance of analyzing clinical and BP 24-h ambulatory separated in studies that have BP as the main outcome. Our study considers this evidence as the clinical BP was more influenced by the effects of NO_3_ of the BRJ by the reduction in SBP and DBP, and the values obtained by the clinical measurement were lower compared to the ambulatory 24-h monitoring.

In the sensitivity analysis, we drew attention to observations in two studies included in the clinical measurement subgroup of SBP. The study by Baião et al. (12) had a high standard deviation, probably because of the limited sample of just 5 participants. When that study was omitted from the meta-analysis, a slight difference was noted in the heterogeneity of the “Clinic” subgroup (*I*^2^ from 62 to 70%) and the overall analysis (*I*^2^ from 67 to 70%), but it did not influence the final result of the meta-analysis on SBP, which decreased to −4.81 mmHg (95% CI: −8.82 to 0.80) in favor of NO_3_. The study by Kapil et al. (16) revealed a reduction in SBP that differed from other studies, which could be because of the large volume of supplementation (250 mL/day), different from the dose that other studies used (70–14 0mL/day). By withdrawing the study from the meta-analysis, the SBP scores in favor of the intervention decreased to −2.73 mmHg (95% CI: −4.85; −0.61). Statistical heterogeneity (*I*^2^) and the test of the difference between subgroups (Chi^2^) reached 0% and, thus, we reiterate the robustness and data reliability in favor of the intervention. The DBP results did not show alterations in any of the sensitivity analysis scenarios.

The meta-analysis published by Siervo et al. (22) with a mixed sample of healthy and hypertensive patients concluded that NO_3_ triggered a reduction in SBP of −4.4 mmHg, but DBP did not change significantly. The study's intervention by Siervo et al. (22) differs from our study in that it includes NO_3_ from BRJ and other sources (e.g., nitrate salts). Despite this, the data corroborated our findings. The meta-analysis by Bahadoran et al. (2), evaluating the effects of BRJ-derived NO_3_ in a mixed population of patients with and without arterial hypertension, demonstrated a mean reduction of −3.55 mmHg for SBP. Although the study population of Bahadoran et al. (2) differs from ours, the outcome was comparable to our study and similar to that of Siervo et al. (22). In contrast, the study by Bahadoran et al. (2) found a significant decrease in DBP at −1.32 mmHg, while in our metaanalysis and the study by Siervo et al. (22) this result was not achieved.

The studies included in our review had a more extended intervention period when compared to those published in the review by Siervo et al. (22) and hence, the nitrate-rich BRJ may represent an effective strategy for the prevention of cardiovascular complications caused by high blood pressure levels in the short and medium-term. Yet, for BRJ NO_3_ to be applied as a long-term intervention, RCTs with a duration of >60 days are still needed. The study by Kapil et al. (16), which had the most prolonged duration, with 60 days of intervention and 250 mL of BRJ with NO_3_, revealed a more significant reduction in SBP and DBP than other studies. The result of this RCT strengthens the data published by Bahadoran et al. (2), in which the intervention of the BRJ with NO_3_ > 14 days and with a greater volume (>140 mL) tended to reduce SBP and DBP more intensely. Then again, we do not yet know the long-term safety of NO_3_ use and, consequently, we suggest caution when extrapolating these findings, as we need evidence to prove the long-term safety of NO_3_ use (25).

The results achieved with this review reinforce the effects of NO_3_ from BRJ as a vital therapeutic adjuvant for the management and control of arterial hypertension in studies of up to 2 months of intervention. Studies with a prolonged treatment time are still needed to ensure that the NO_3_ of the BRJ is effective in lowering BP for a long period and, as an alternative, being able to reduce the rates of coronary heart disease, stroke, heart failure, renal failure, and all-cause mortality in hypertensive patients (26).

Bearing in mind that hypertension has a global prevalence, NO_3_ of the BRJ may have a great contribution to the prevention of complications of arterial hypertension. As a result of its high morbidity, in the USA alone, hypertension is responsible for a cost of US$131 billion per year for its control and complications from the disease (27). It is also imperative to emphasize that adopting nutritional strategies together to control hypertension can enhance the clinical importance of dietary interventions. The diet approach to stop hypertension (DASH), which advocates increased consumption of fruits, vegetables, and low daily fat intake, reduces SBP and DBP by −5.2 mmHg and −2.6 mmHg, respectively (28). Replacing sodium chloride with potassium chloride also has positive contributions in reducing −5 mmHg in SBP and −2 mmHg in DBP (29).

This review is a pioneer in demonstrating the effects of NO_3_ from the BRJ on BP parameters of an exclusive population of hypertensive patients, along with evaluating clinical and outpatient BP values separately and together. The results are promising and support results already revealed in other meta-analyses commenced on patients with other health features (2, 24).

It should be stated that the included studies had important methodological limitations to be considered. Standardization techniques in clinical and outpatient measurements to obtain BP values were not reported. Clinical trials have a limited number of participants who have different characteristics and conditions (e.g., young and old, taking medication, other chronic diseases). Also, the experimental protocols of the studies have different designs (e.g., crossover, parallel). Despite all these restrictions, we underline that there is no evidence to date to refute the benefits of nitrate-rich BRJ for the cardiovascular health of hypertensive patients.

### Perspectives

The aspects identified in this meta-analysis will provide some guidelines for future studies to improve the understanding of nitrate-rich BRJ effects on the cardiovascular health of individuals with hypertension. Since the number of studies with a particular sample of hypertensive patients is still limited, further research is desired to confirm the results achieved in this meta-analysis. A metaregression did not apply due to the small number of studies included in the review. The Cochrane Handbook (8) recommends a minimum number of 10 studies, and we were not able to stratify the effects of specific participants' characteristics (e.g., other morbidities) in the primary studies included in this meta-analysis. Clarifications on the safety of using NO_3_ chronically are still required to confirm the benefits of NO_3_ on long-term cardiovascular health. Bearing in mind that the BP values of hypertensive patients are differently influenced by way of measurement in which they are obtained, we suggest that future studies obtain data by the auscultatory (clinical) and ambulatory 24-h monitoring method together. Impending studies may need to analyze the hypotensive effects of other beetroot compounds compared to NO_3_.

In conclusion, this systematic review with meta-analysis supports that the NO_3_ of the BRJ is an effective intervention in reducing the SBP of patients with arterial hypertension in interventions of up to 2 months duration.

## Data Availability Statement

The raw data supporting the conclusions of this article will be made available by the authors, without undue reservation.

## Author Contributions

CJRB and AP performed conduction of experiments, performed the statistical analysis, wrote introduction, methods, results, and discussion sections. AS and BG improved interpretation analysis and reviewed the manuscript. DG draft the manuscript and improved interpretation analysis and reviewed English Grammar and Spelling. CRBJ and VV supervised the study, draft the manuscript, and gave final approval for the version submitted for publication. All authors contributed to the article and approved the submitted version.

## Funding

This study was financed in part by the Coordenação de Aperfeiçoamento de Pessoal de Nível Superior – Brasil (CAPES) – Finance Code 001.

## Conflict of Interest

The authors declare that the research was conducted in the absence of any commercial or financial relationships that could be construed as a potential conflict of interest.

## Publisher's Note

All claims expressed in this article are solely those of the authors and do not necessarily represent those of their affiliated organizations, or those of the publisher, the editors and the reviewers. Any product that may be evaluated in this article, or claim that may be made by its manufacturer, is not guaranteed or endorsed by the publisher.
