# Supplementary figures and images for: Nitrate Derived From Beetroot Juice Lowers Blood Pressure in Patients With Arterial Hypertension: A Systematic Review and Meta-Analysis
Source: Front Nutr. 2022 Mar 15;9:823039. doi: 10.3389/fnut.2022.823039 (PMC8965354; doi:10.3389/fnut.2022.823039)

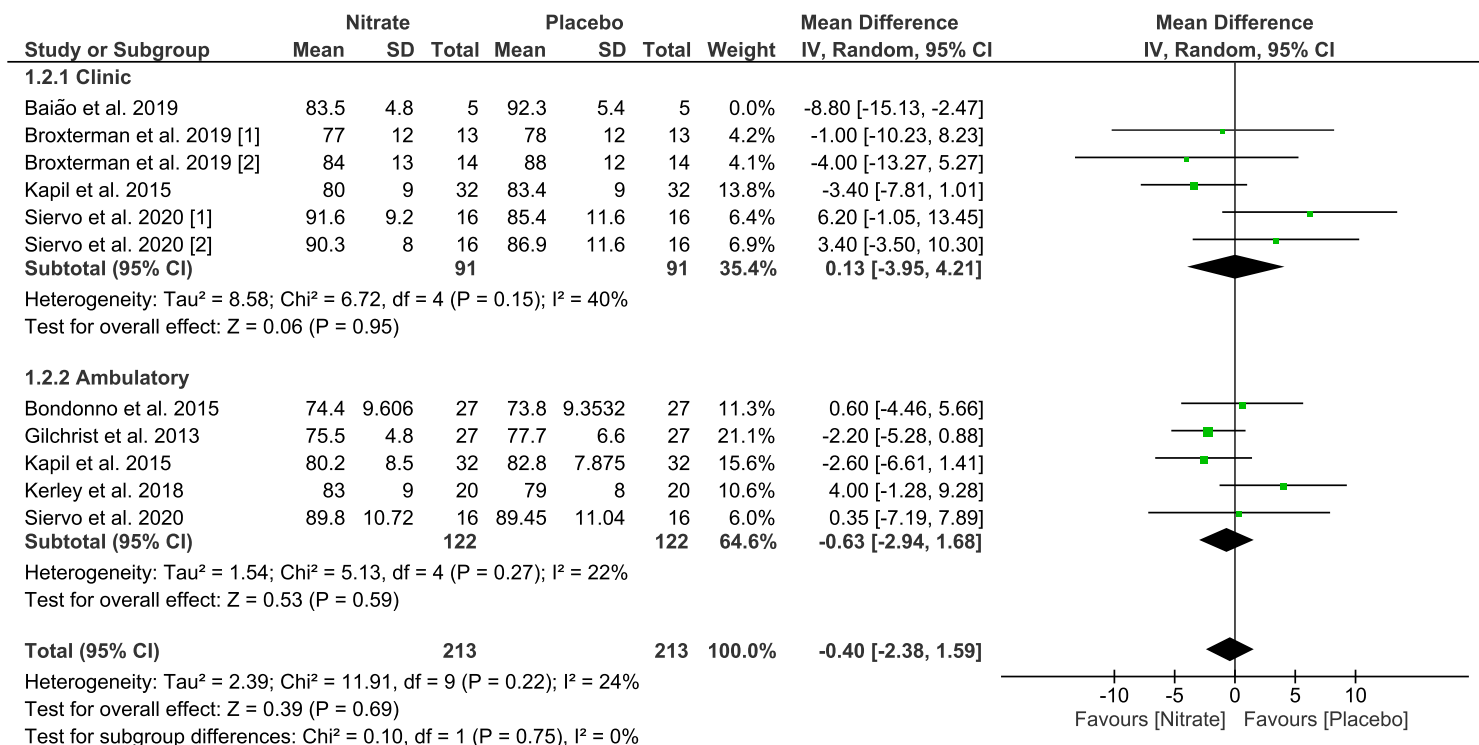

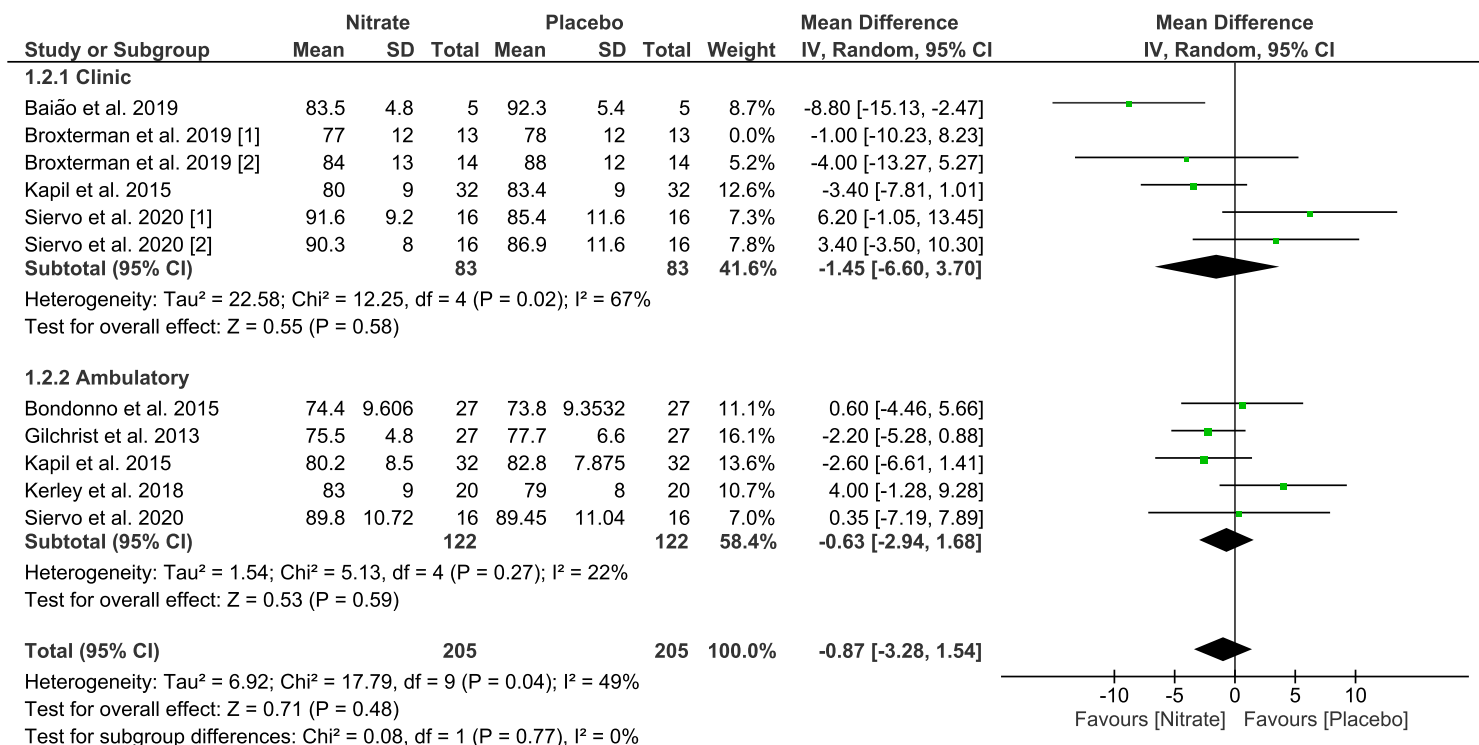

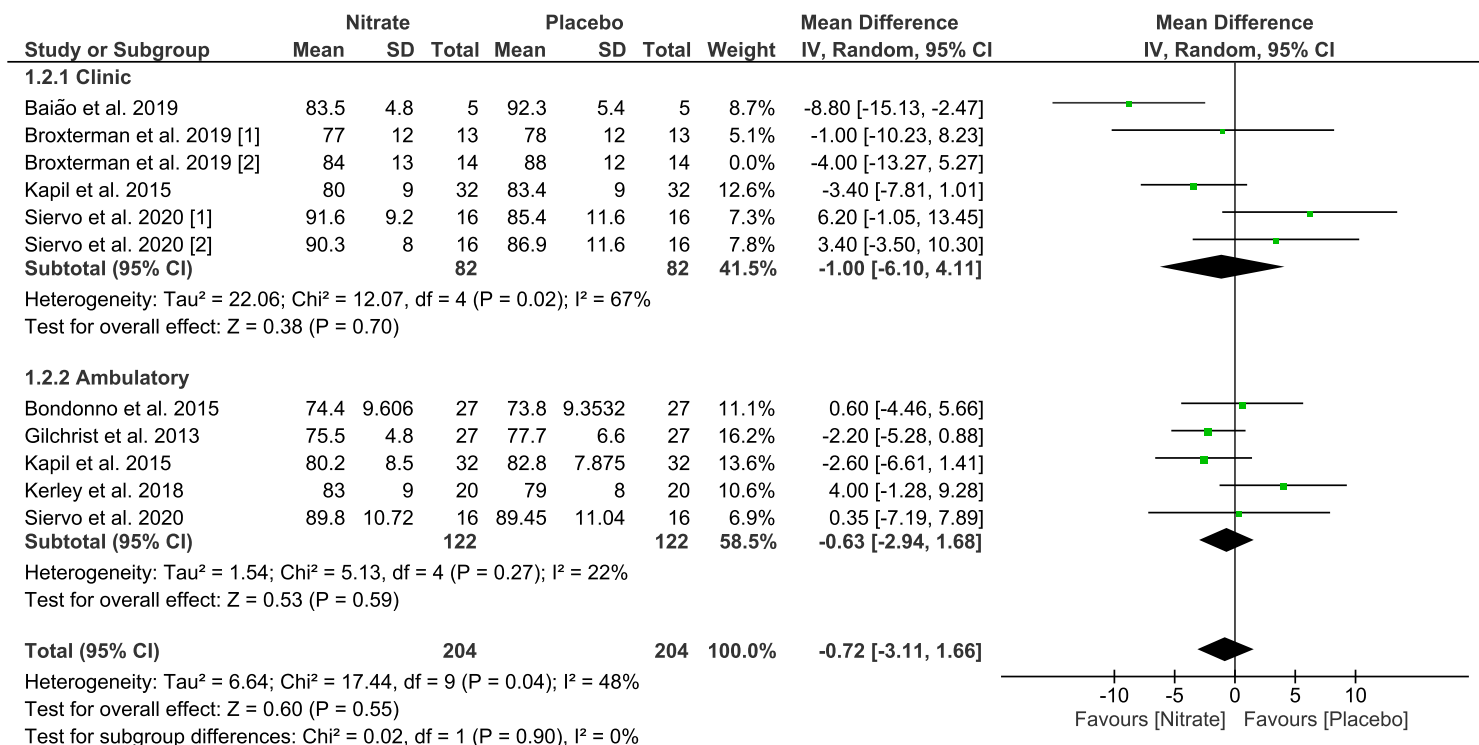

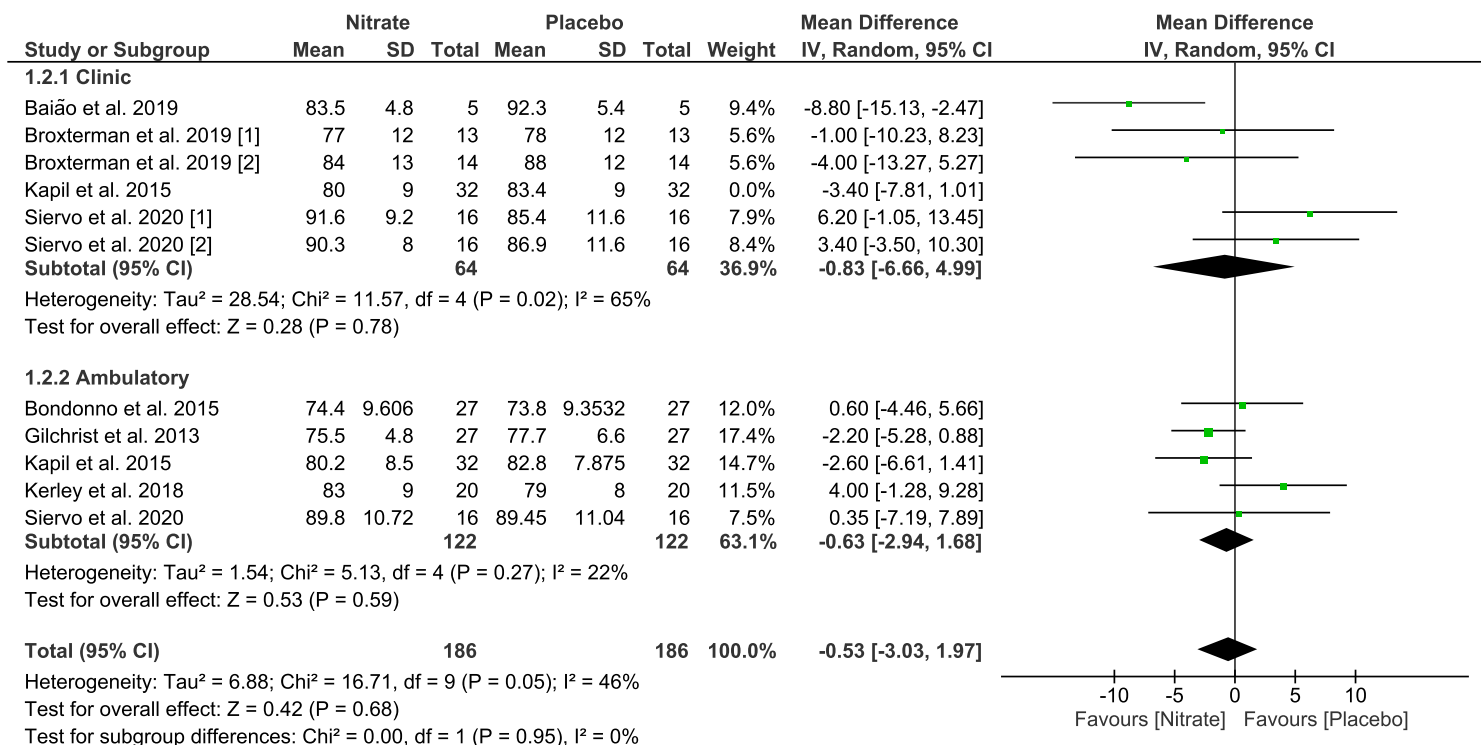

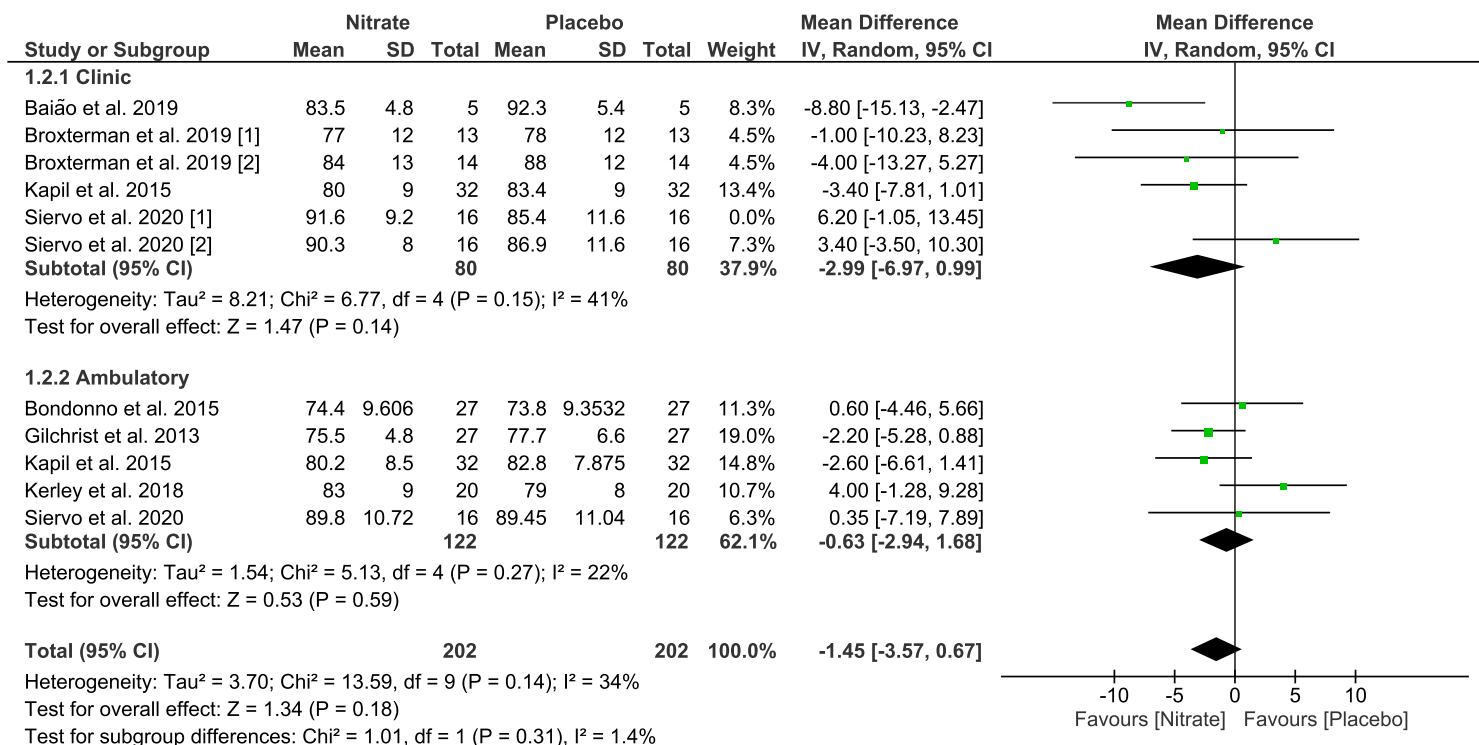

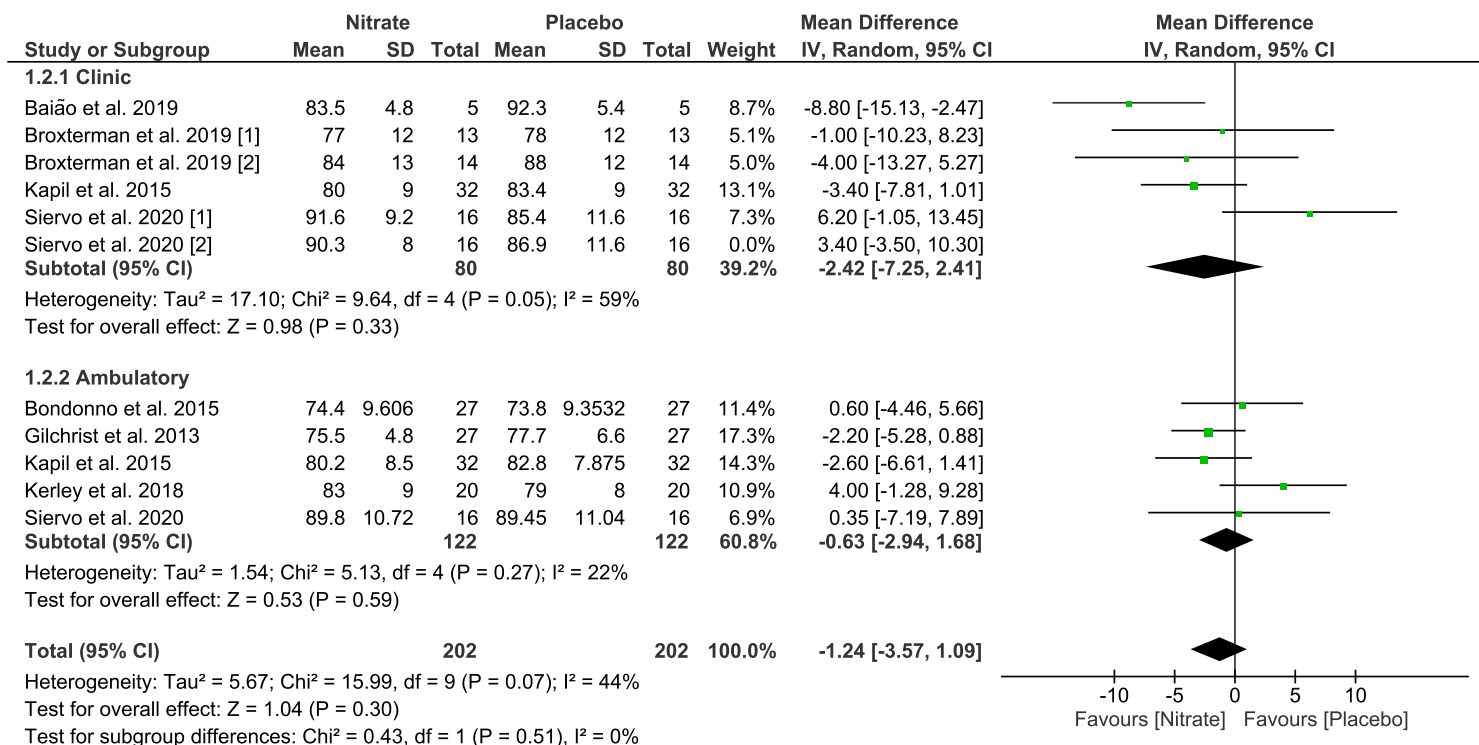

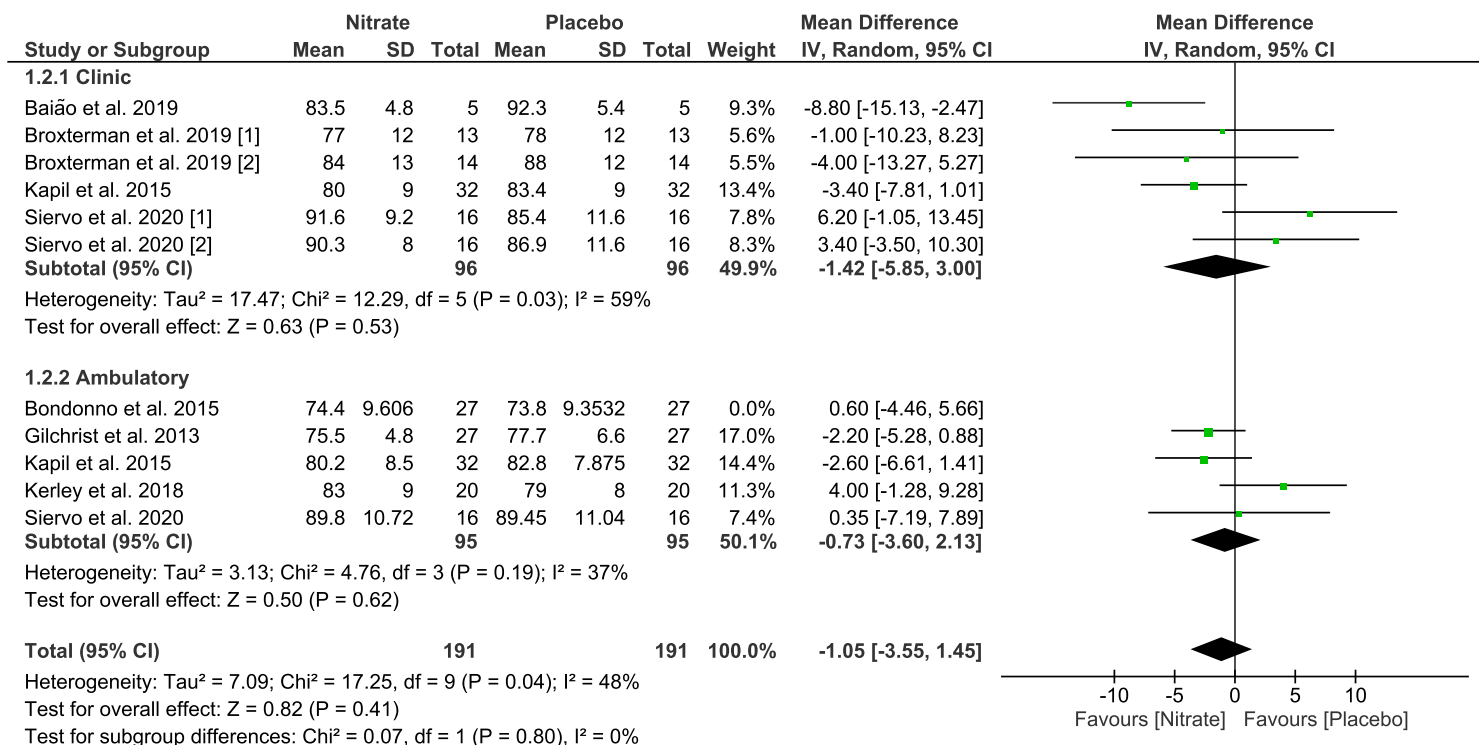

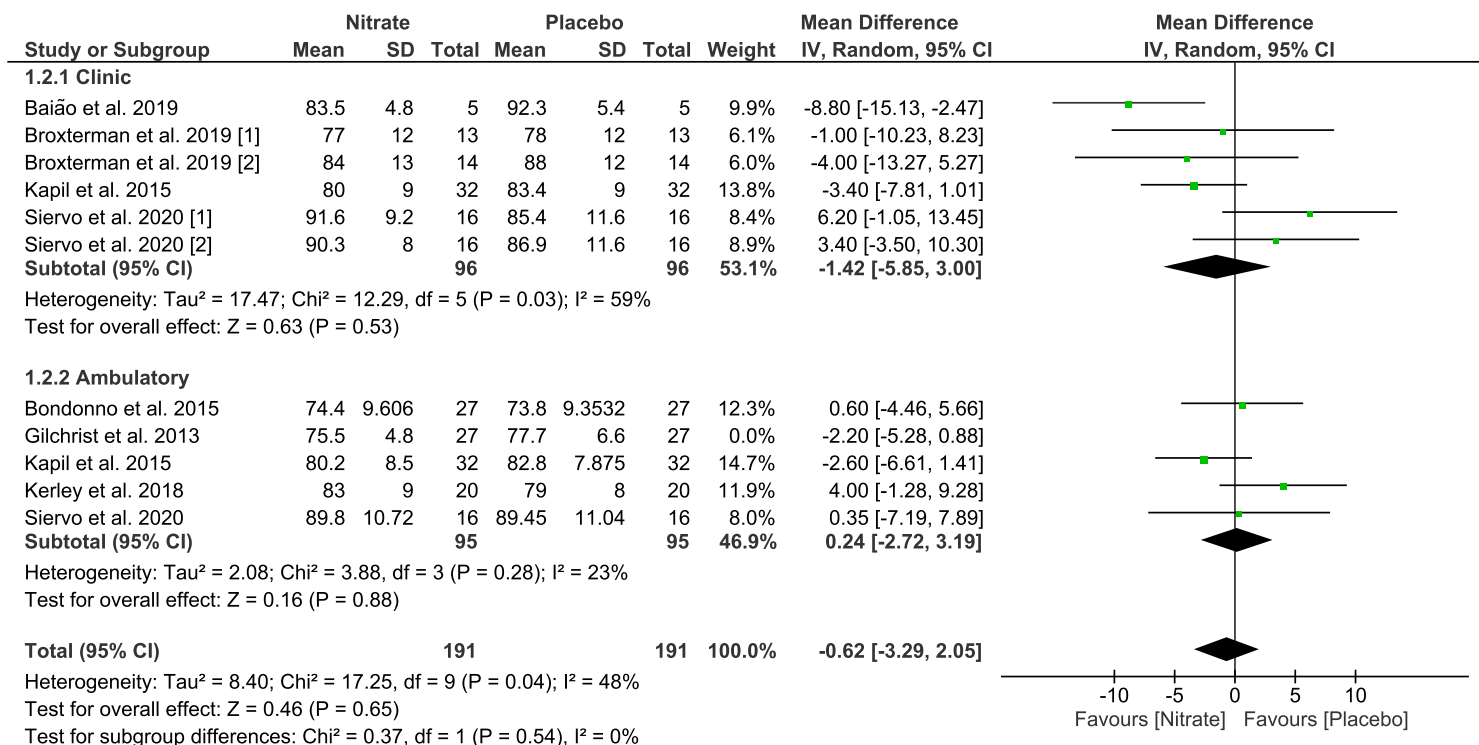

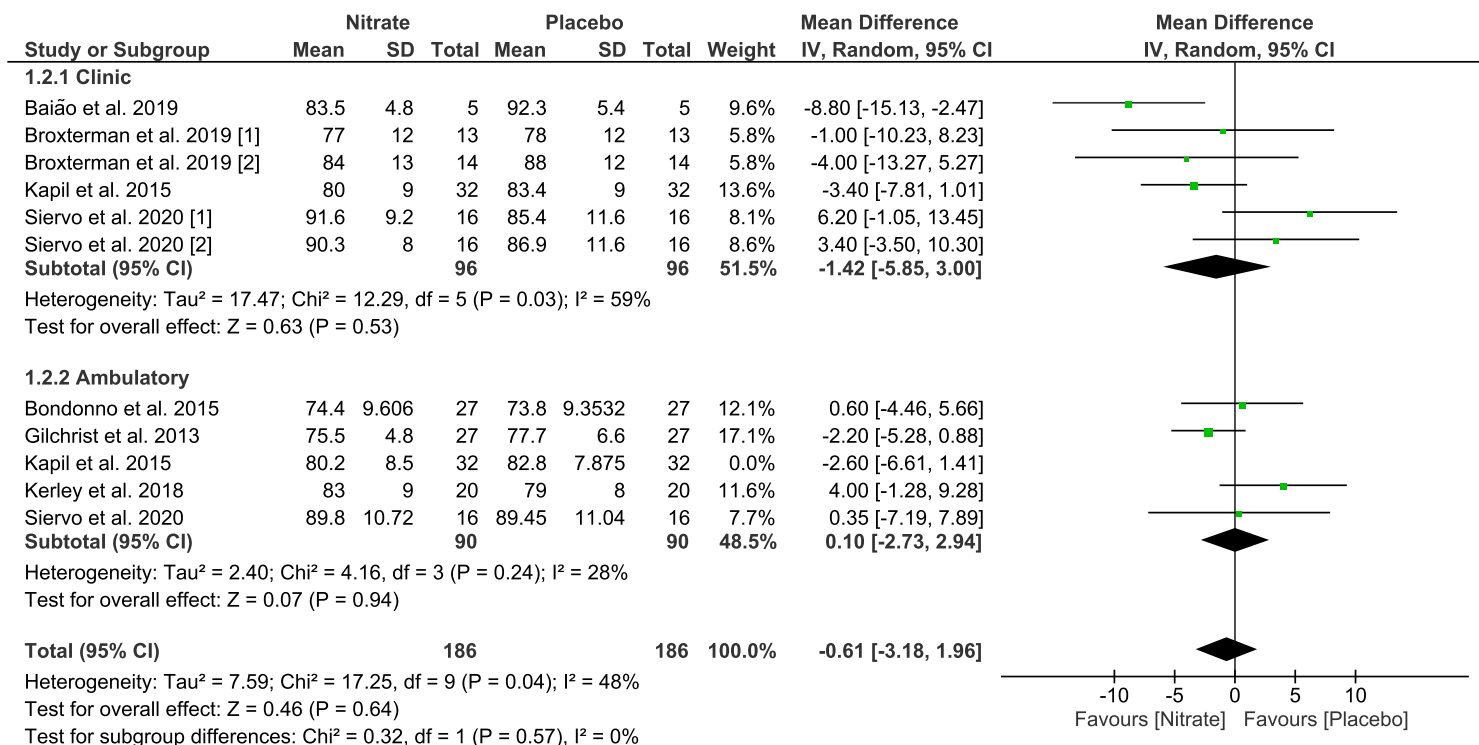

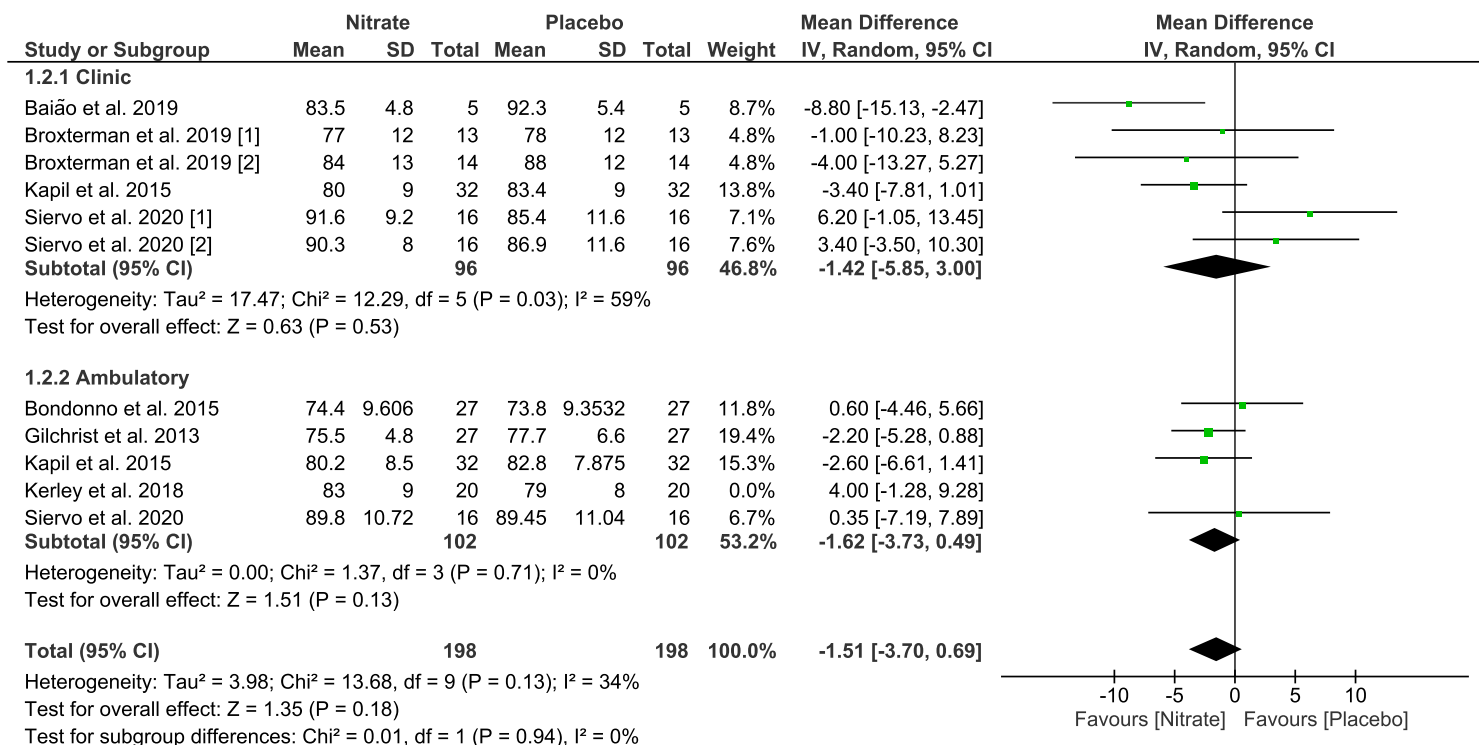

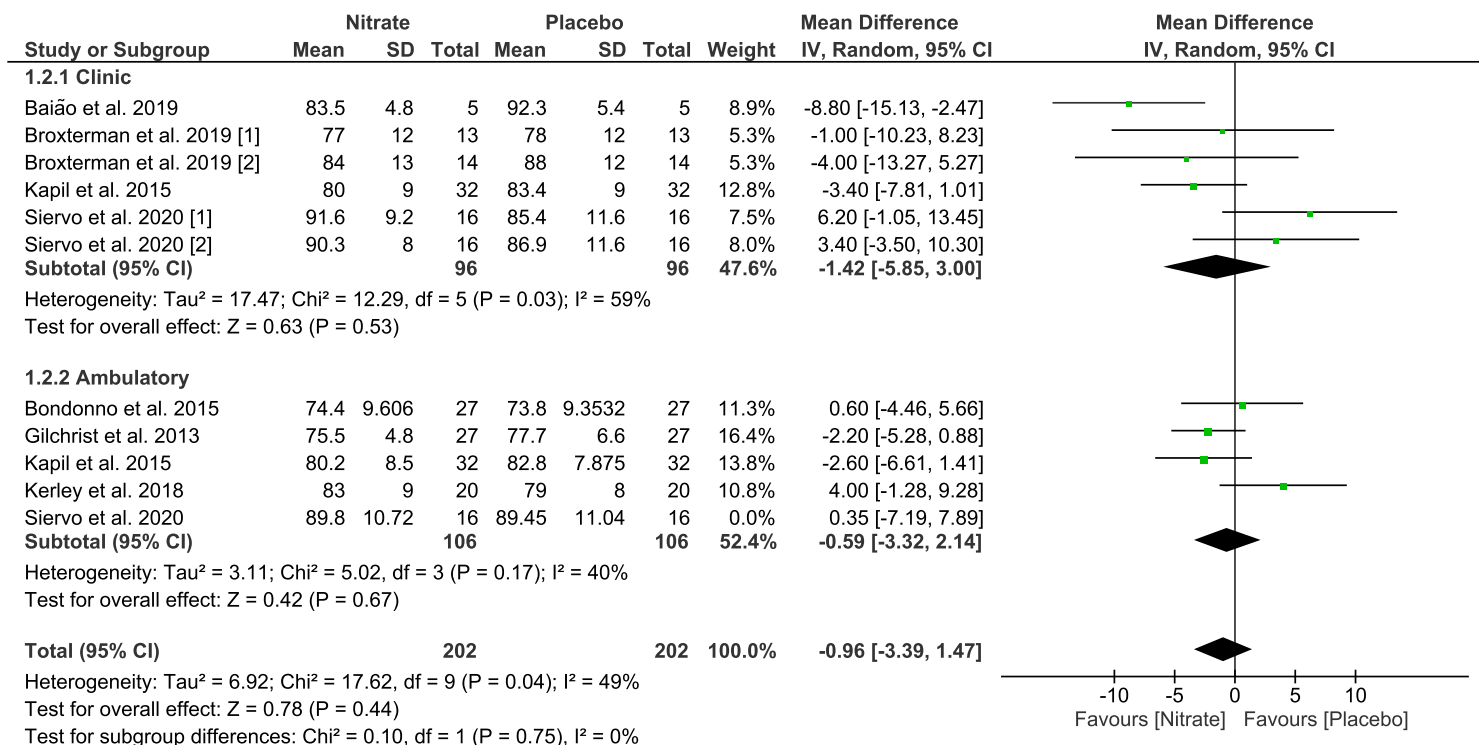

Supplement: Supplementary file 1 [file Data_Sheet_1.PDF]

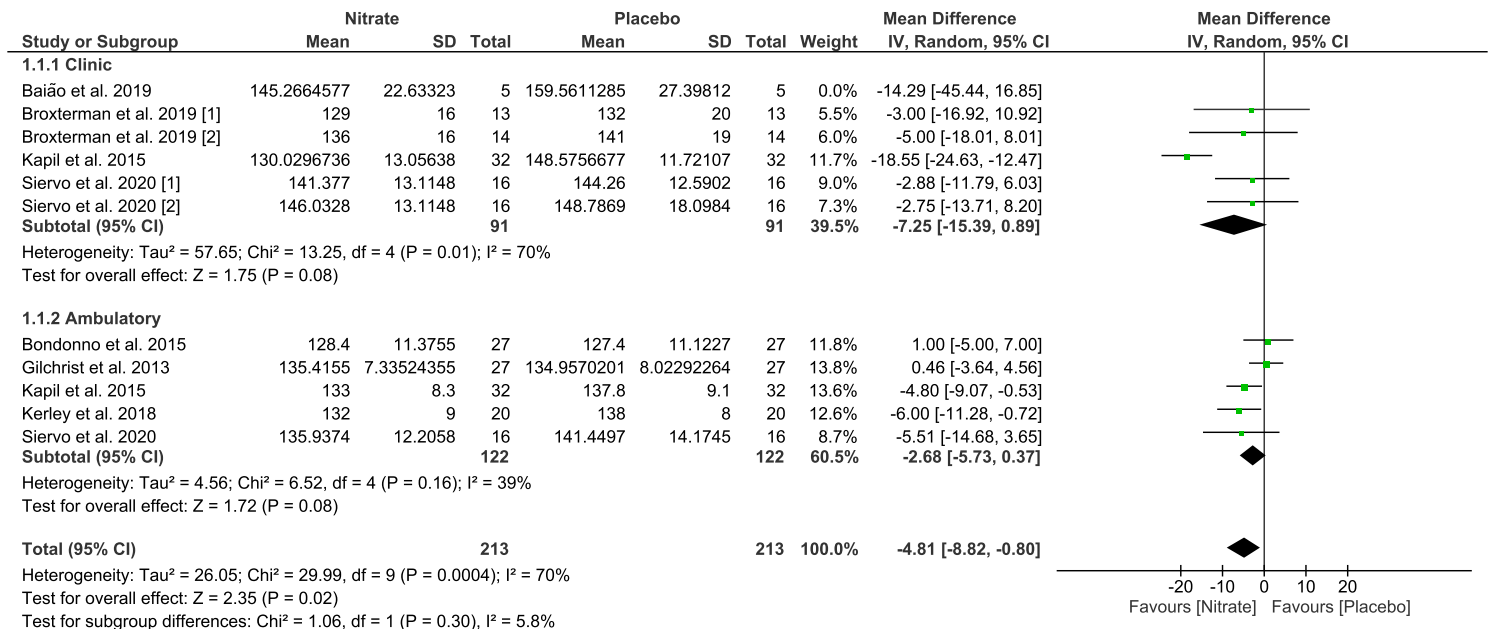

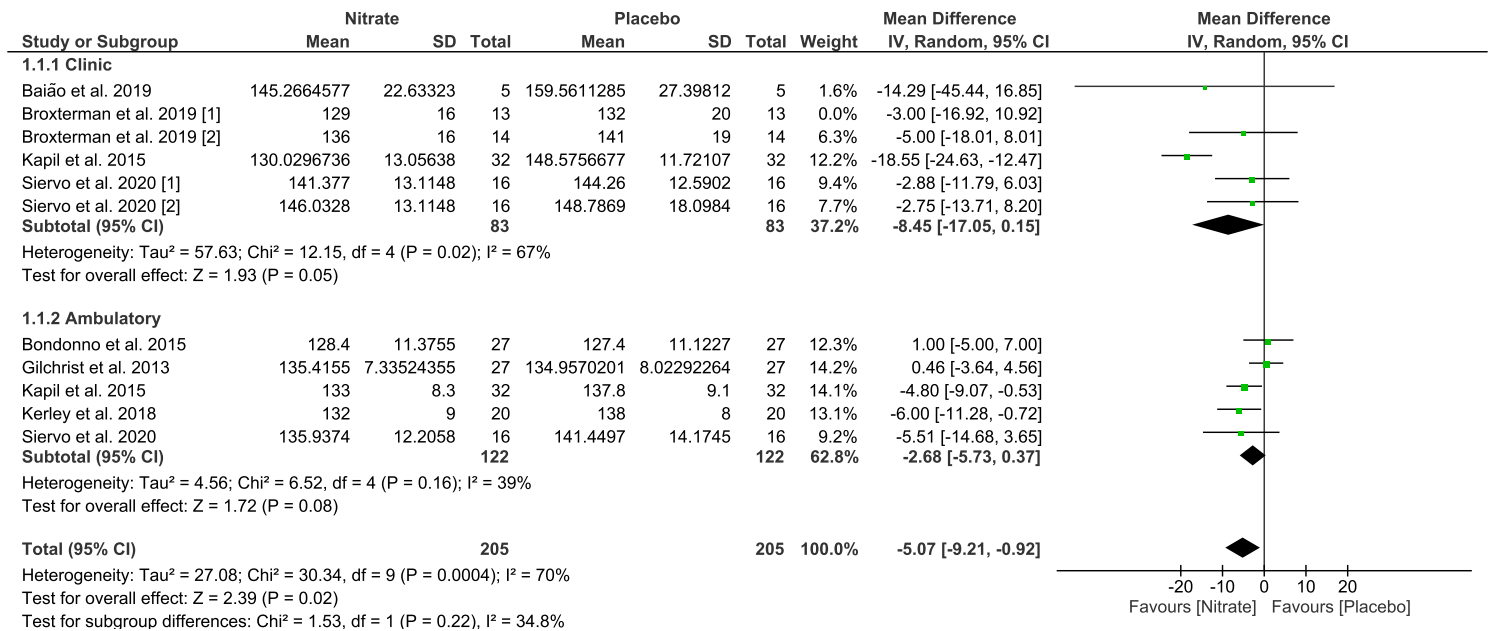

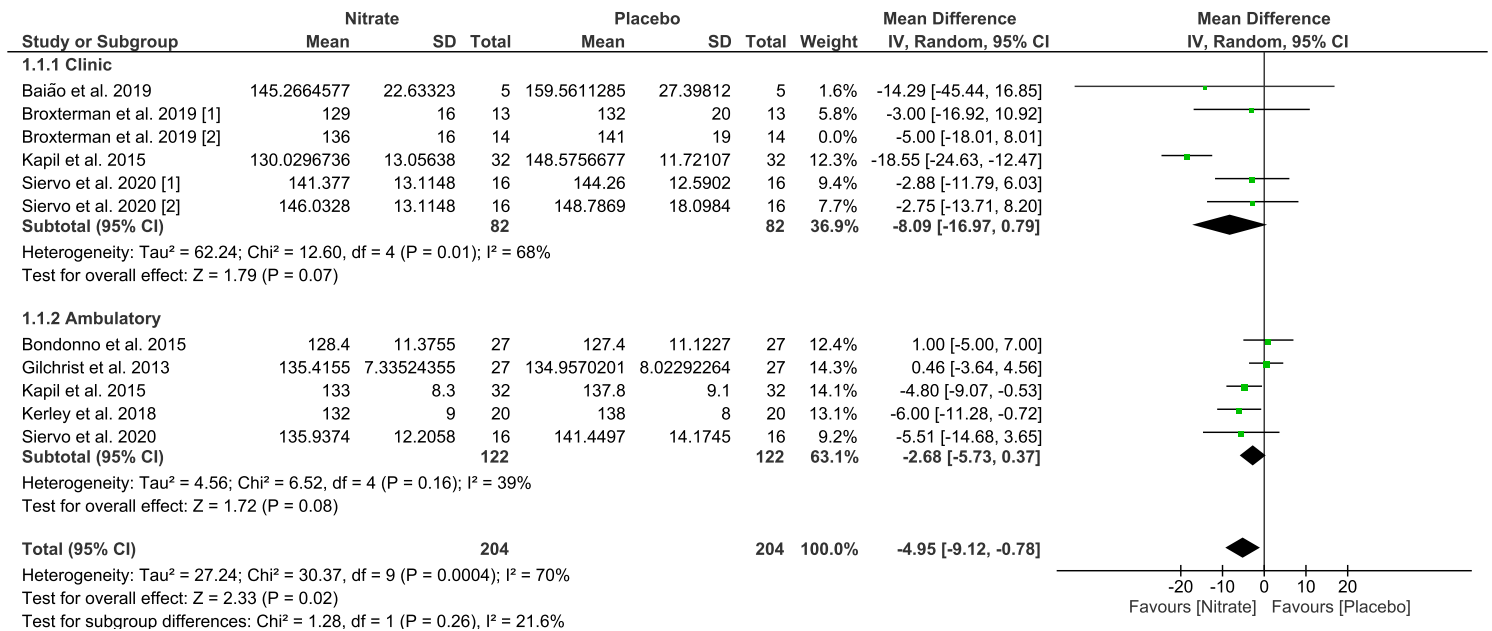

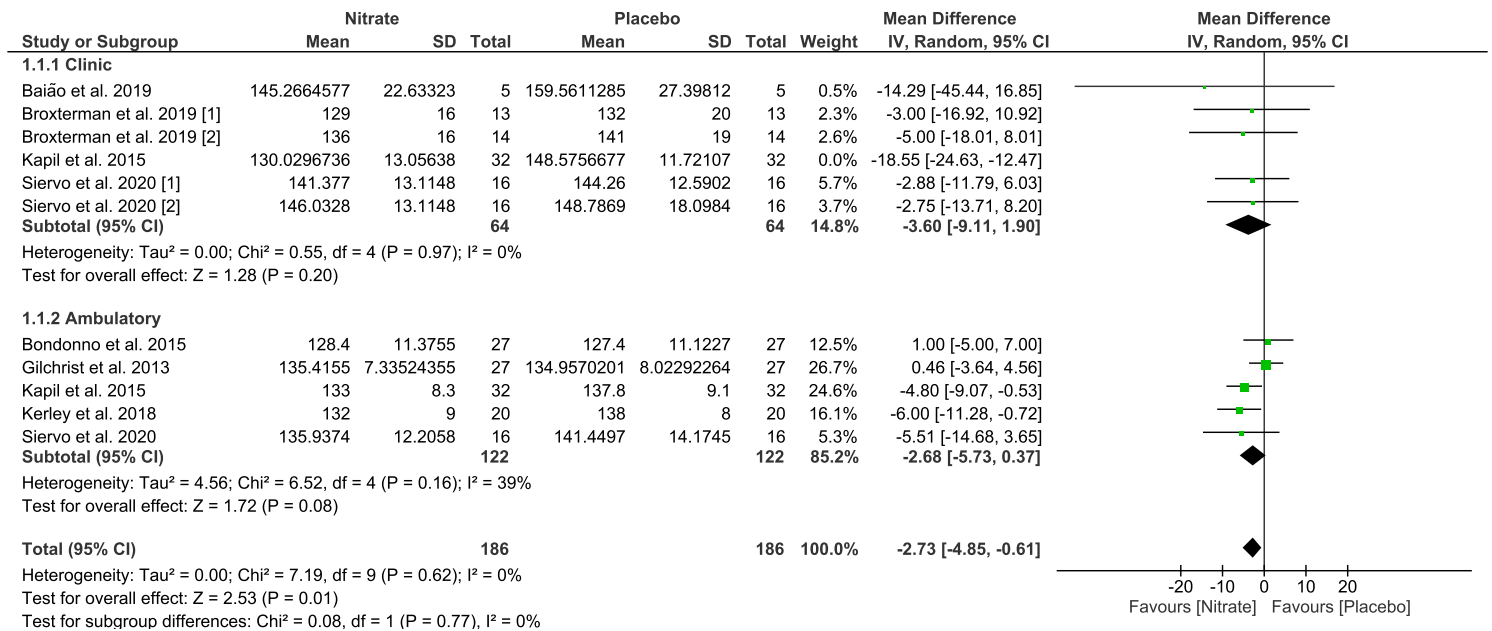

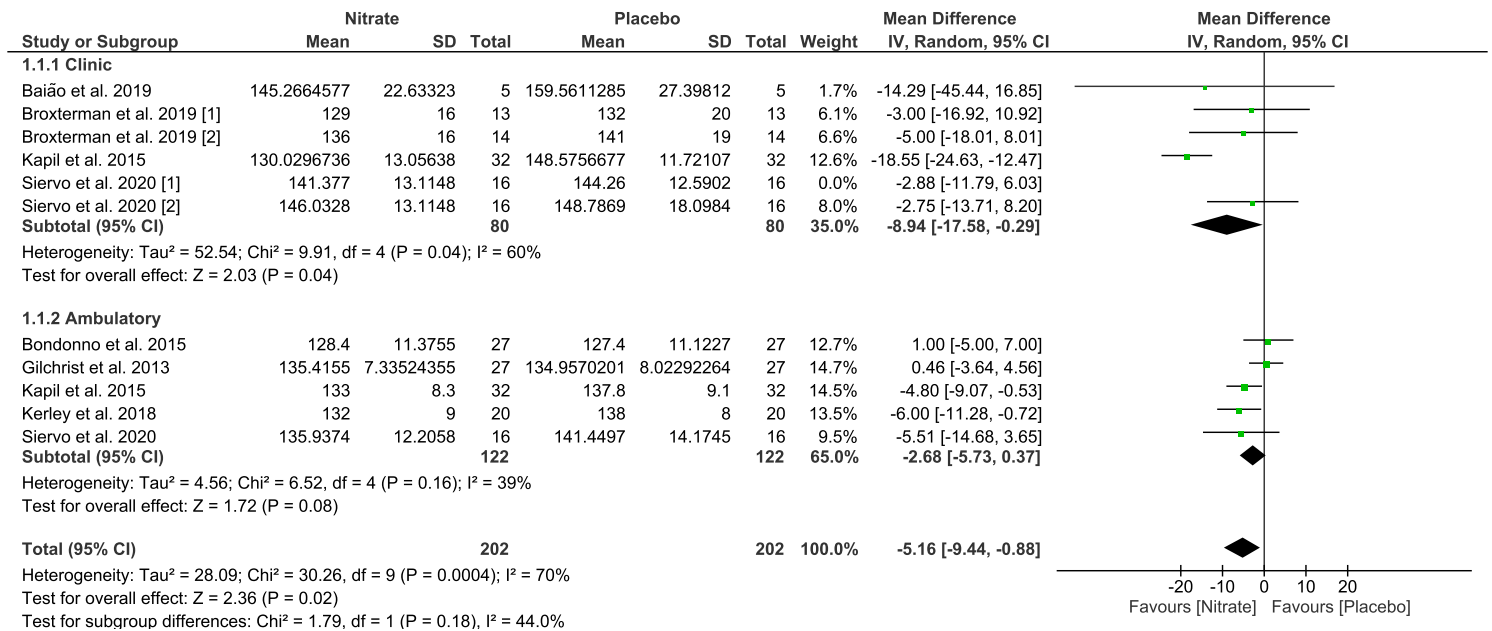

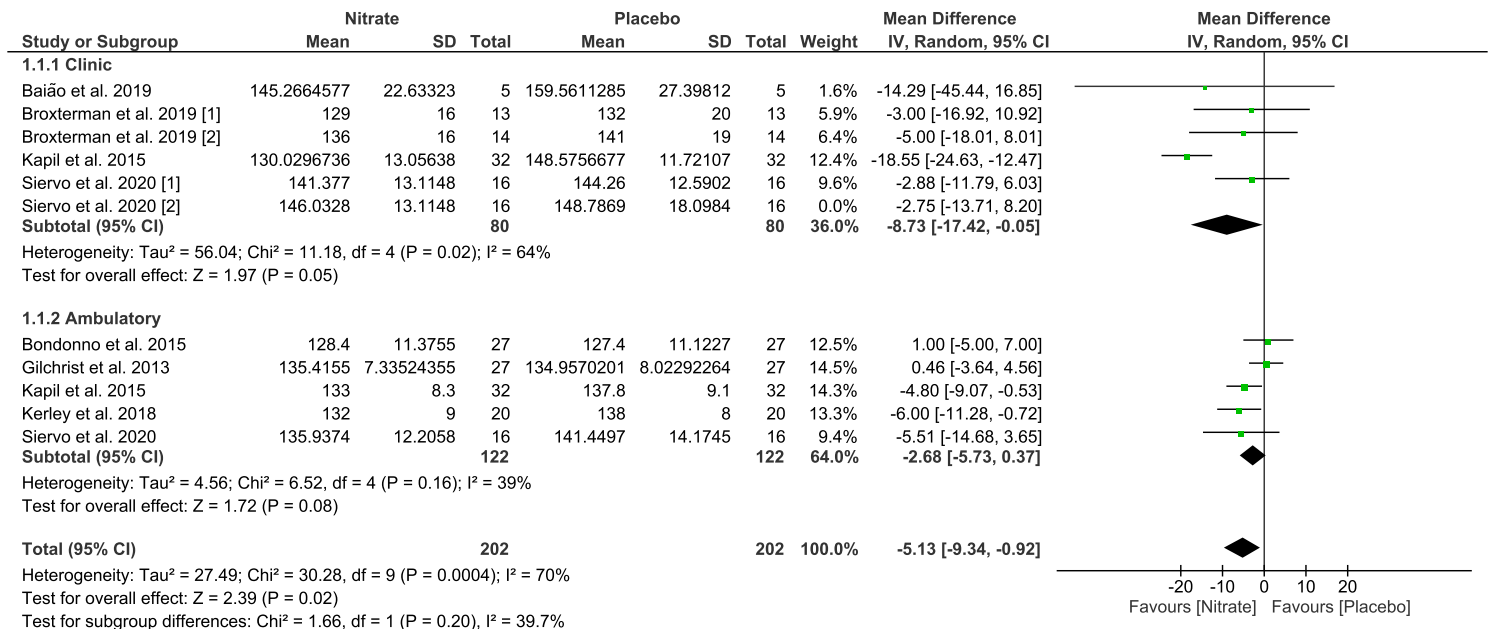

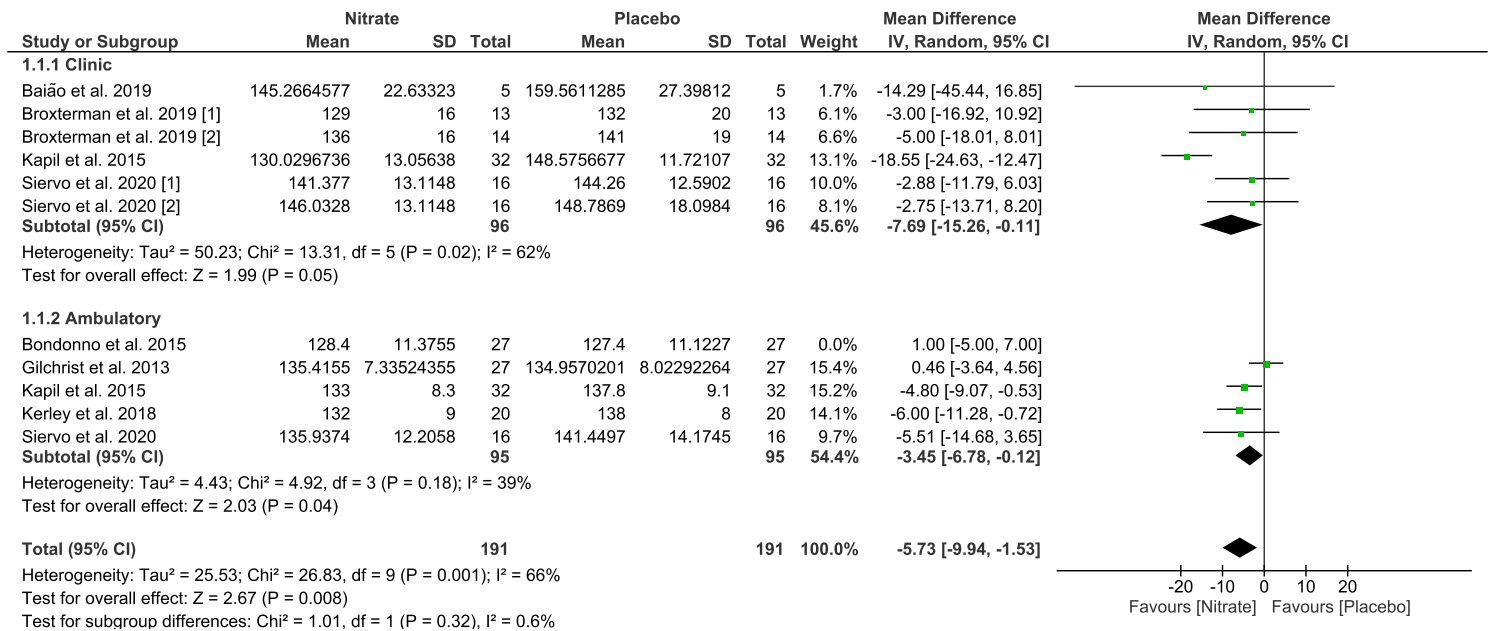

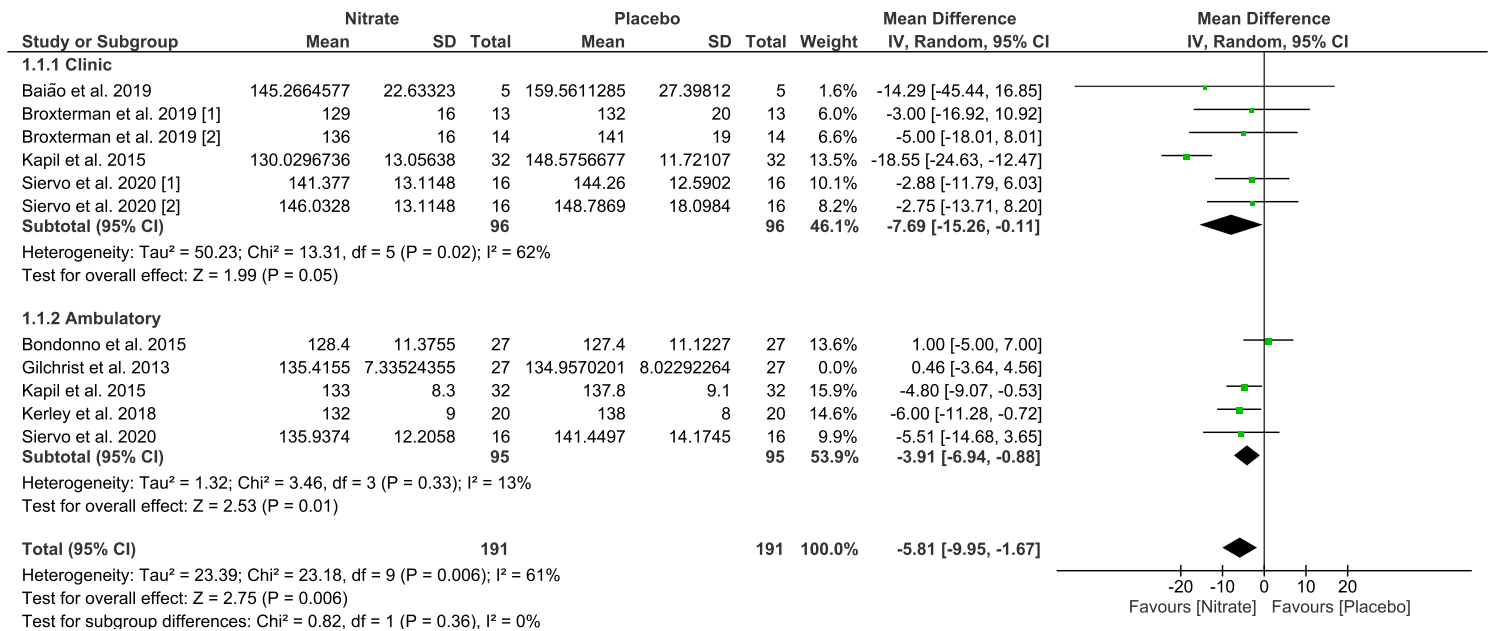

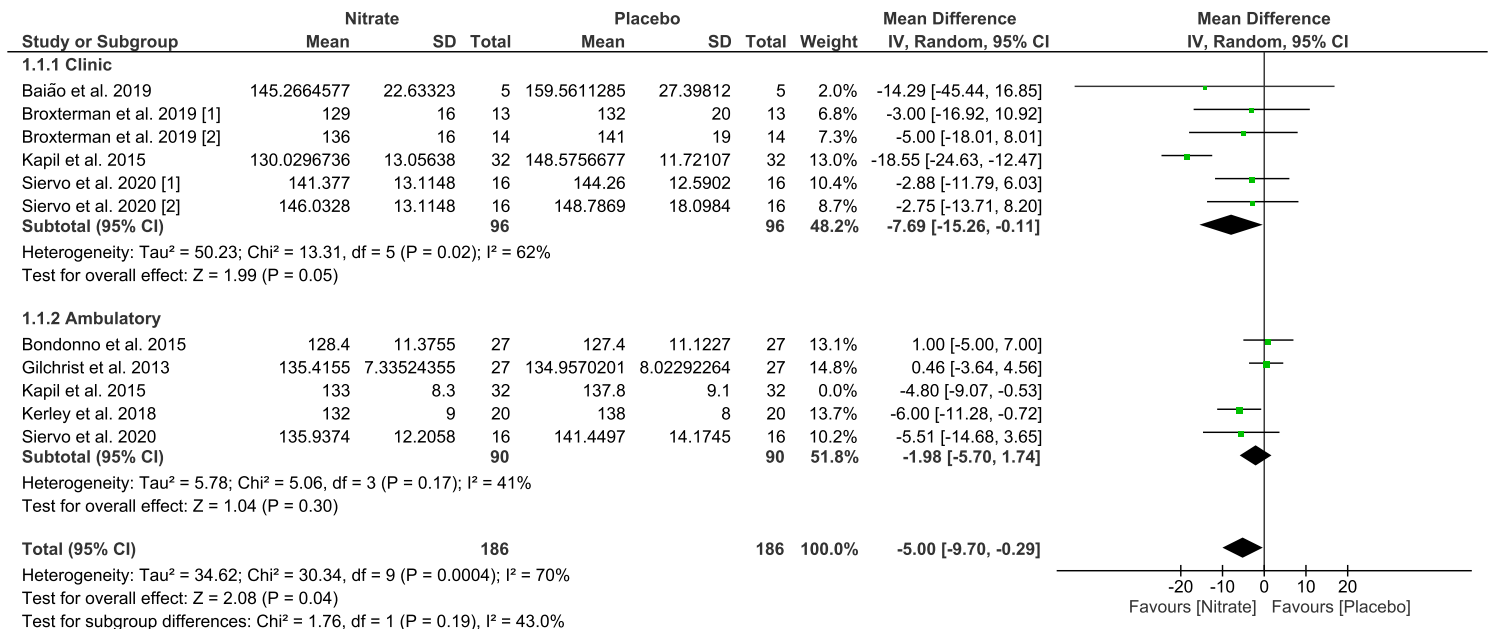

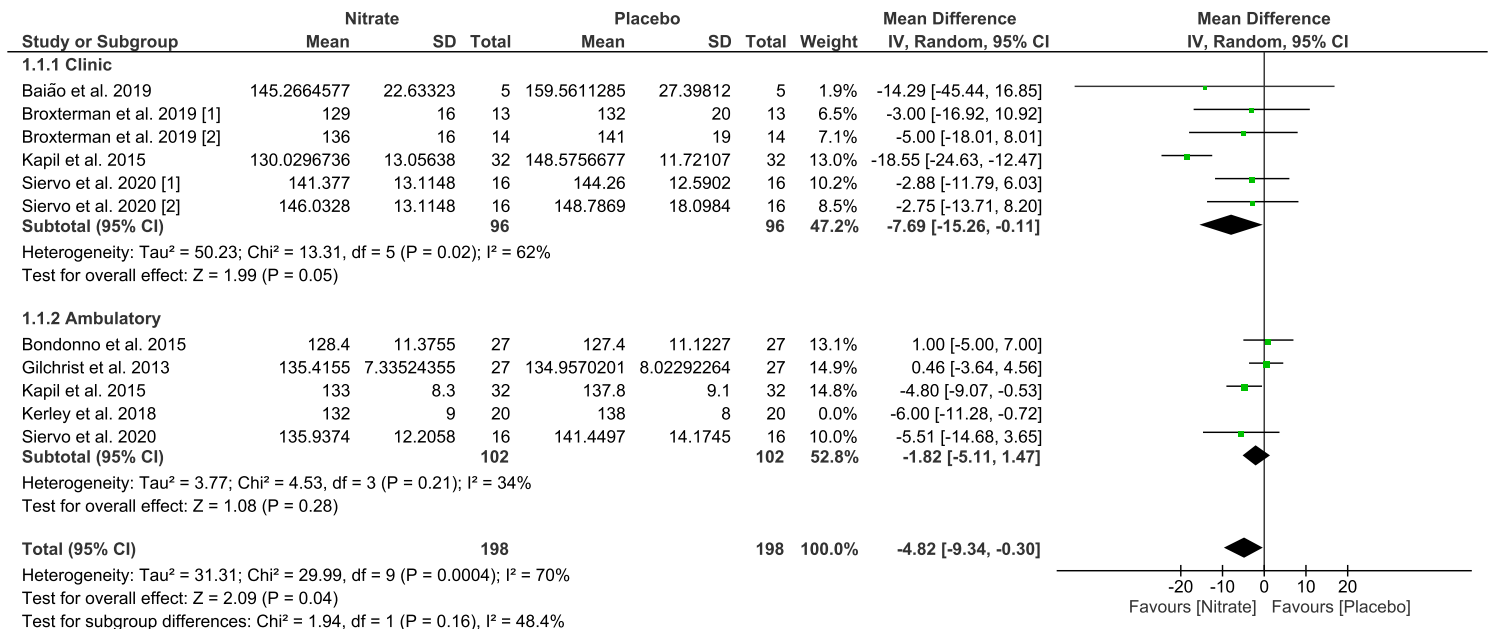

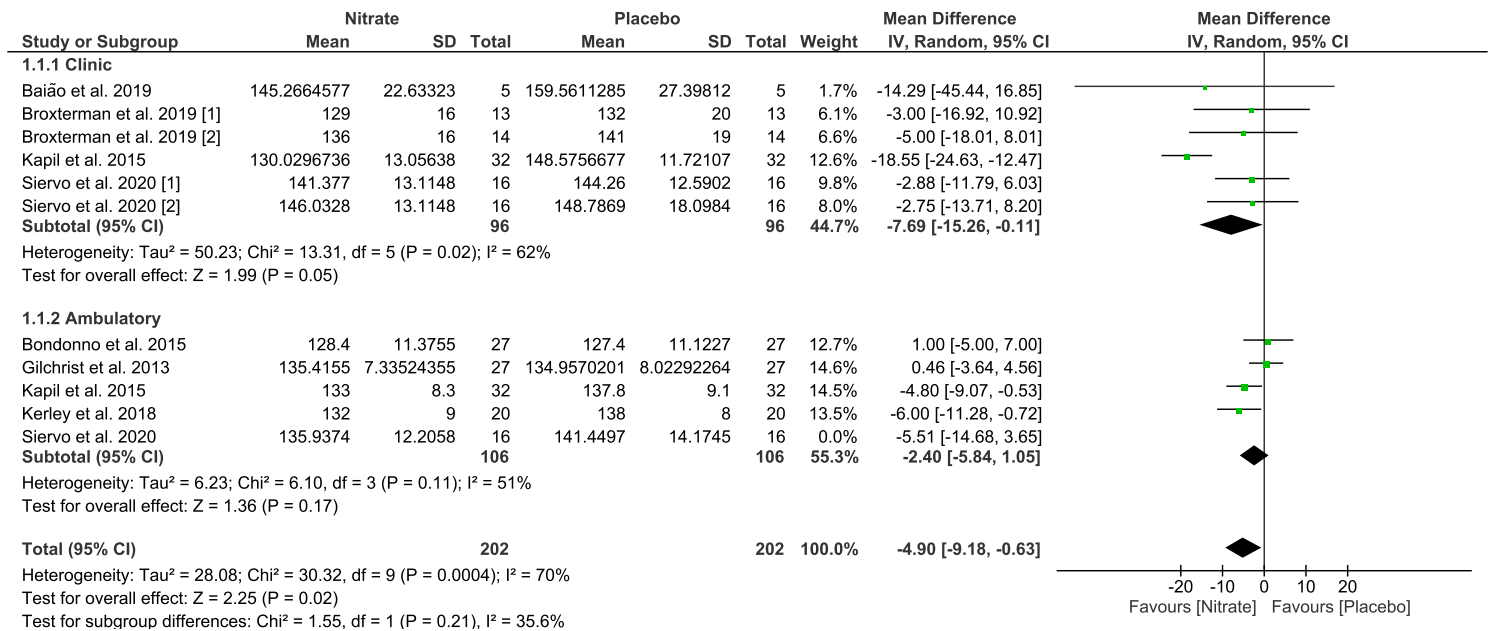

Supplement: Supplementary file 2 [file Data_Sheet_2.PDF]
